# Red Phosphorus by Atomic Layer Deposition

**DOI:** 10.1021/acs.nanolett.6c01078

**Published:** 2026-04-28

**Authors:** Raul Zazpe, Jaroslav Charvot, Jhonatan Rodriguez-Pereira, Jiří Váňa, Luděk Hromádko, David Pavliňák, Jan Mistrik, Michal Kurka, Jan M. Macak, Filip Bureš

**Affiliations:** a Center of Materials and Nanotechnologies, Faculty of Chemical Technology, 48252 University of Pardubice, Studentska 95, 532 10 Pardubice, Czech Republic; b Central European Institute of Technology, Brno University of Technology, Purkynova 123, 612 00 Brno, Czech Republic; c Institute of Organic Chemistry and Technology, Faculty of Chemical Technology, University of Pardubice, Studentska 95, 532 10 Pardubice, Czech Republic; d Institute of Applied Physics and Mathematics, Faculty of Chemical-Technology, University of Pardubice, Studentska 95, 532 10 Pardubice, Czech Republic

**Keywords:** Atomic layer deposition, Phosphorus synthesis, Thin films, Nanoparticles, Metal-free semiconductor

## Abstract

Herein, a new synthetic procedure for red phosphorus (RP) by atomic layer deposition (ALD) is presented. The deposition of RP thin films was obtained by combining tin tetrachloride and home-synthesized tris­(trimethyltin)­phosphide. The ALD window was identified between 175 and 200 °C, and the self-limiting nature of the ALD process was verified. The RP thin films deposited on Si/SiO_2_ wafer, soda lime glass, and carbon paper were extensively characterized by different techniques, revealing that the phosphorus was deposited exclusively as elemental RP without any concomitant phosphorus species. The assessment of the optical properties revealed an optical bandgap of ≈1.9 eV confirming the semiconducting nature of the RP thin films. To gain valuable insight into the ALD reaction mechanism, density functional theory calculations suggested a plausible mechanism that combined ligand exchange and redox steps. This work extends the portfolio of ALD precursors and fills the gap in the missing deposition of elemental P.

## Graphical abstract

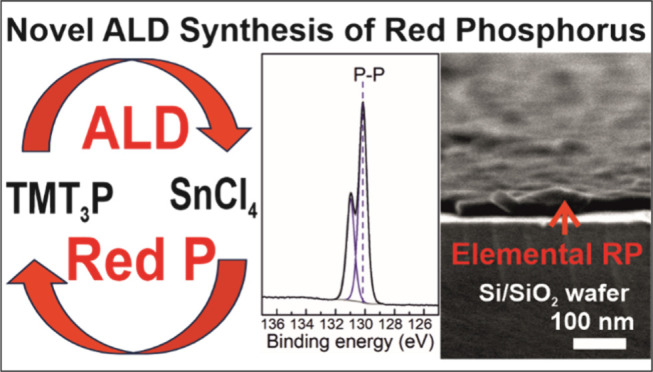

Red phosphorus (RP) has attracted great interest recently since the discovery of its semiconducting properties with a band gap ranging from 1.70 to 2.0 eV emerging as an interesting alternative to conventional photocatalysts, such as TiO_2_.^1^ Compared to the black allotrope, metal-free semiconducting RP photocatalyst is more abundant and low-cost, with fascinating inherent properties and promising perspectives to address both sustainable energy and environmental remediation. RP has shown enormous potential for visible light driven water splitting,^1^ microbial inactivation,^2^ and photocatalysis of N_2_ fixation,^3^ and CO_2_ reduction.^4^ Its visible light response reveals a strong tail at approximately 700 nm,^1^ and when it is combined with UV-active materials, its optical absorption capacity can be effectively extended.^5^ In parallel, the benefits of RP in energy storage applications have also been recognized due to the relatively small volume change during (dis)­charging processes, its ability to alloy with all the main alkali ions, low redox potential, high theoretical capacity, and excellent cycling stability.^6^ Nevertheless, RP suffers from a high recombination rate for photogenerated electron–hole pairs and short charge carrier lifetime during illumination.^7,8^ The fabrication of RP-based high surface area heterostructures with rational heterojunction design could both promote the spatial separation and mitigate the recombination rate and offer an enlarged number of surface-active sites, enhanced mass transfer ability, and higher adsorption capacity, boosting the photocatalytic activity efficiency. Therefore, the conformal and uniform deposition of photocatalytic material for attaining an optimal design of high surface area heterostructures is highly desirable yet very demanding.

Nowadays, there are different methods for the synthesis of RP, including hydrothermal,^9^ solvothermal,^10,11^ precipitation methods,^12^ high temperature solid-state reaction,^13,14^ vacuum deposition,^1^ ball milling,^15^ and chemical vapor deposition.^16,17^ Here, it must be noted that in most synthetic approaches, the starting material is bulk commercial RP.^18^ Few exceptions reported the fabrication of RP by thermal decomposition and *in situ* phosphorization reaction using sodium hypophosphite.^19,20^ However, these synthetic methods suffer from some limitations, impeding them from fulfilling the requirements of conformality and uniformity and limiting the control over the thickness of thin films. In contrast, atomic layer deposition (ALD) due to inherent properties offers unique advantages over conventional RP synthetic methods enabling homogeneous, high quality, and conformal coating, along with unparallel subnanometer thickness control. Compared to PVD and CVD, ALD offers superior step coverage and superior conformality at lower operating temperatures, respectively. As to the ALD advantages over time demanding multistep solvothermal and hydrothermal methods, they are a single step deposition process, simplifying significantly RP synthesis and avoiding the use of solvents.

ALD is a well-established deposition technique based on alternating gas-surface chemical reactions of self-limiting nature between precursor and substrate. The chemical reaction proceeds via alternate exposure of the substrate surface to gas phase precursor molecules chemically tailored to react saturatively with the surface groups.^21,22^ Due to these unique features, ALD is an ideal candidate for the design and fabrication of nanomaterials or their functionalization via coatings of secondary materials.^23^

The synthesis of different P-based materials by ALD has been closely related to the development of suitable P precursors. In the early stages the P ALD precursors suffered from a low vapor pressure, a high deposition temperature, and/or corrosive/toxic byproducts. It triggered an intense effort toward the design and development of suitable P precursors to replace those originally used. As a result, trimethyl phosphate (TMP) and related derivatives were explored toward the synthesis of a wide number of metal phosphates (M_*x*_(PO_4_)_*y*_) by thermal and plasma ALD approach as recently reviewed by L. Henderick et al.^24^ The family of ALD P-based materials was enlarged by the synthesis of metal phosphorus oxynitrides,^25,26^ and several metal phosphides, such as GaP,^27^ InP,^28^ and CoP,^29^ using a limited number of P precursors, namely tris­(dimethylamino)­phosphine, tertialybutylphosphine (TBP), and phosphine (PH_3_). However, unlike tris­(dimethylamino) phosphine, the latter two (TPB and PH_3_) are difficult to handle since they are highly toxic and potentially pyrophoric. Recently, a new suitable P compound, tris­(trimethyltin)­phosphide (TMT_3_P) has been successfully developed as an ALD precursor and yielded the very first ALD synthesis of TiP.^30^ Thus, in the last few years the ALD P-based materials family has been significantly enlarged, yet it is not complete, as elemental P is still elusive.

Thus, the ALD synthesis of elemental RP is presented for the first time in this work by combining TMT_3_P and SnCl_4_ precursors. The synthesis of RP (described in detail in the Supporting Information) was exploited within a temperature range between 150 and 250 °C. Carbon paper, Si/SiO_2_ wafer, and soda lime glass were used as substrates to evaluate their effect on the RP growth. The effect of the deposition temperature was also explored by a set of ALD processes applying different deposition temperatures: 150, 175, 200, 225, and 250 °C. The thickness of the resulting thin films deposited on Si/SiO_2_ wafers at different temperatures were measured by cross-sectional SEM images, and the results exhibited an ALD window between 175 and 200 °C characterized by a growth rate of ≈0.055 nm/cycle (Figure 1a). The RP films exhibited a strong growth rate dependence on the deposition temperature, as shown in Figure 1a and Figure S1, where the optical picture of soda lime glass substrates coated with 650 ALD cycles at different deposition temperatures is shown. The substrates showed different reddish colors due to the differences in thickness of the RP thin film deposited caused by different growth rates observed as a function of the deposition temperature in Figure 1a. Thus, the lower deposition temperature (150 °C) drove a lower growth rate that can be directly linked to a thermal energy limitation of the reaction. Meanwhile, the growth rate drop observed at higher deposition temperatures (225 and 250 °C) could be originated by different causes occurring concomitantly, such as desorption of the precursors molecules from the surface before the chemical reaction leading to a decrease in the areal density of reactive groups, and/or partial sublimation of the deposited elemental phosphorus. The higher the temperature, the more intense the desorption effect. The linear relation between the thickness of the resulting RP thin films and the number of ALD cycles was verified by a set of ALD processes at 200 °C applying different numbers of cycles on Si/SiO_2_ wafers (Figure 1b). Representative optical picture of soda lime glass substrates and cross-sectional SEM images of the RP thin films upon different numbers of ALD cycles can be seen in Figure S2 and S3, respectively. The soda lime glass substrates in Figure S2 exhibited a gradual increase of the reddish color intensity with an increasing number of ALD cycles arising from thickening RP thin film. The self-limiting feature of the reaction that verifies the ALD nature of the process was assessed by a set of ALD processes run at 200 °C applying different doses of each precursor while keeping the dose of the other precursor constant (Figure 1c,d). The TMT_3_P doses were 350, 500, and 750 ms (keeping the SnCl_4_ dose constant at 75 ms), and the SnCl_4_ doses were 35, 75, 125, and 175 ms (keeping the TMT_3_P dose constant at 500 ms). The results revealed a self-limiting regime for TMT_3_P doses ≥ 500 ms and SnCl_4_ doses ≥ 75 ms verifying the ALD nature of the process showing a growth per cycle of ≈ 0.055 nm/cycle.

**Figure 1:**
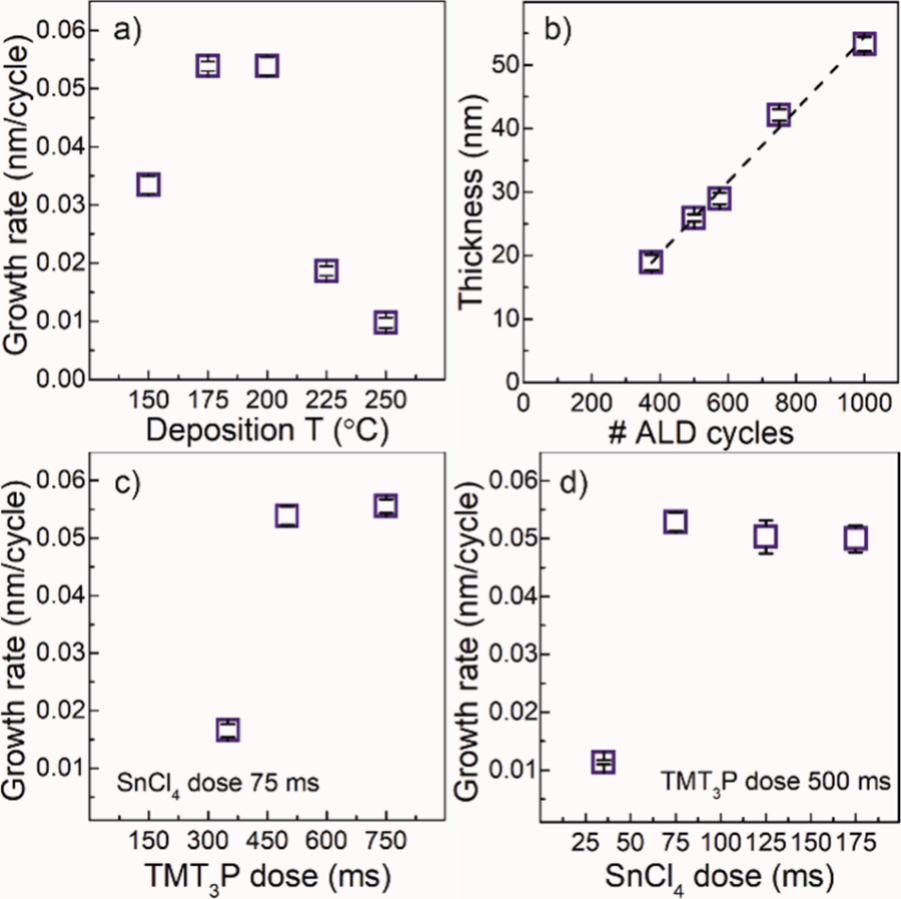
(a) RP thin film growth rate dependence on the deposition temperature on Si/SiO_2_ wafer applying a 500 ms dose of TMT_3_P and 75 ms dose of SnCl_4_. (b) Characteristic linear relation exhibited by a typical ALD process between RP thin film thickness versus the number of ALD cycles obtained at 200 °C showing a GPC of around 0.05 nm/cycle. The RP growth rate on a Si/SiO_2_ wafer at 200 °C as a function of (c) the TMT_3_P precursor dose and (d) SnCl_4_ dose. Saturation regime was observed at 500 and 75 ms dose values of TMT_3_P and SnCl_4_, respectively.

The morphology of the as-deposited RP was characterized by top view scanning electron microscopy (SEM) and atomic force microscopy (AFM). Top view SEM images revealed a nucleation delay illustrated by the deposition of discrete nanoparticles for a low number of ALD cycles (25), which grow, eventually coalesce, and form a continuous layer on CP (Figure 2) and Si/SiO_2_ wafers (Figure S4) after a higher number of ALD cycles. Longer nucleation delay was observed on CP substrates as compared to that on Si/SiO_2_ wafers, principally originated by a lower number of active surface sites that act as initiation sites, indicating a substrate effect on the RP growth behavior.

**Figure 2:**
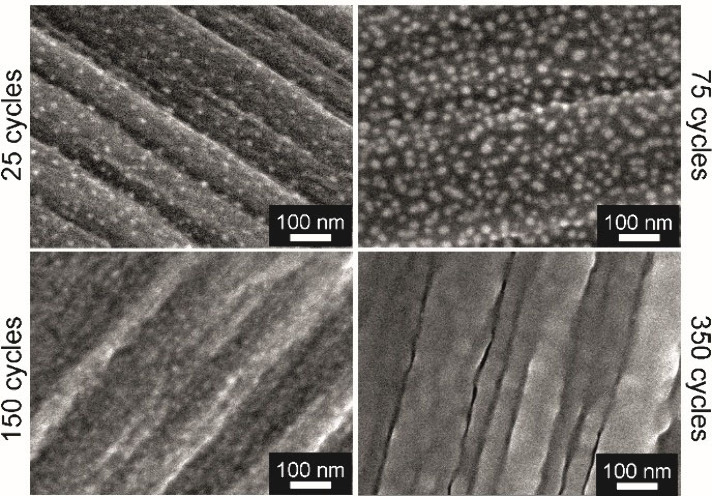
Top-view SEM images illustrate the growth and coalescence of RP nanoparticles on CP to eventually form a continuous RP thin film (deposited at 200 °C).

Atomic force microscopy (AFM) measurements were carried out in order to characterize the roughness of the deposited RP on atomically flat Si/SiO_2_ wafers as a function of the number of ALD cycles (see Figure S5). The RP thin films deposited after different numbers of ALD cycles revealed apparent granular morphology with similar mean roughness values shown in Table S1. In Figure S5 one can observe the nucleation of discrete RP nanoparticles during the early ALD stage (25 ALD cycles), while further growth of these RP nanoparticles upon a higher number of ALD cycles drove them to coalesce, resulting in the formation of a continuous granular thin film.

The crystal structure of the RP thin films was analyzed by grazing incident X-ray diffraction (GI-XRD). The XRD pattern showed two broadened diffraction peaks at ≈13–17° and ≈24–35° (Figure S6) characteristic of amorphous RP and in line with the literature.^20,31^ An additional peak was observed at ≈22° denoted by an asterisk. However, after an extensive survey in the literature, such a peak could not be indexed to either RP allotrope or any P-based and Sn-based species, and the nature of the peak is unknown. The structural analysis of the RP thin films deposited on soda lime glass (see Figure S7) was further complemented by Raman spectroscopy performed using a laser of 532 nm as described in the Experimental Section, Supporting Information. The Raman spectrum exhibited a broad band in the frequency range 350–500 cm^–1^ (see Figure 3) characteristic of amorphous RP. The peaks at 347, 385, and 460 cm^–1^ can be ascribed to the bond bending modes (fundamental mode B_1_), bond bending vibrations (symmetric stretch modes A_1_), and stretching vibrations (degenerate mode E_1_) of amorphous RP respectively, which is consistent with the literature.^32–34^

**Figure 3:**
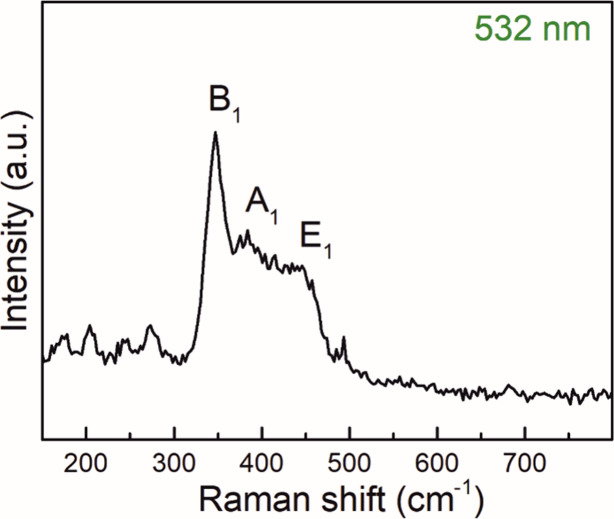
Raman spectrum obtained using a 532 nm laser from RP thin film grown on soda lime glass after 650 ALD cycles.

The surface chemical composition of the RP thin films was characterized by X-ray photoelectron spectroscopy (XPS). Figures S8 and S9 show the XPS survey spectra of RP obtained at 200 °C after 650 ALD cycles on Si/SiO_2_ wafers and soda lime glass, respectively, revealing the presence of P, C, and O and traces of Cl and Sn. The corresponding elemental composition on Si/SiO_2_ wafers and soda lime glass is shown in Table S2 and Table S3, respectively. According to density functional theory (DFT) calculations described ahead, the most plausible reaction mechanism between both precursors involves a ligand exchange reaction, suggesting that the residual content of Sn and Cl originates from an incomplete ligand exchange reaction, while O was mainly ascribed to the oxidation due to exposure to air before XPS analysis as O-free precursors were used. The effect of deposition temperature on the chemical composition was assessed by RP thin films deposited at 150, 175, 200, 225, and 250 °C on Si/SiO_2_ wafers after 650 ALD cycles. The corresponding peak fitted high-resolution (HR) P 2p XPS spectra obtained are shown in Figure 4 along with the spectrum obtained from the commercial RP powder used as a reference. The peak fitting of the HR P 2p XPS spectra with its characteristic spin–orbit splitting of P 2p_3/2_ and P 2p_1/2_ at ≈129.9 and ≈130.8 eV respectively allowed the identification of the presence elemental P (P–P). Despite the presence of O, it was not related to the formation of phosphate, whose presence would originate peaks at ≈133–134 eV.

**Figure 4:**
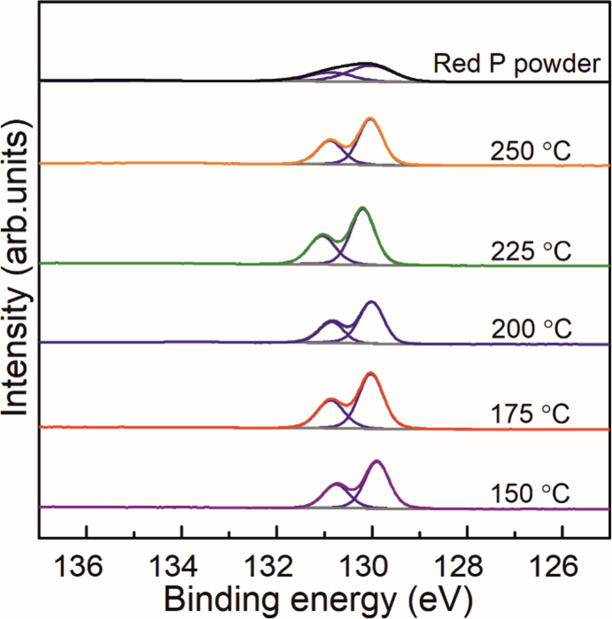
High-resolution P 2p XPS spectra from RP thin films after 650 ALD cycles on Si/SiO_2_ wafers at different deposition temperatures.

The elemental composition of the RP deposited at different temperatures is shown in Table S2. The results showed higher residual content of Sn and Cl at low deposition temperatures (150 and 175 °C) which significantly decreased at higher deposition temperatures (200, 225, and 250 °C). The presence of Sn and Cl was mainly associated with partial retention of ligands on the surface after each ALD cycle primarily due to the above-mentioned thermodynamic limit of the metal precursor, which requires a minimum thermal energy to initiate and complete the ligand exchange reaction. Thus, the presence of the residual content of Sn and Cl was constrained to the surface, yet deposition as impurities could not be ruled out. Interestingly, the results also indicated that the different deposition temperatures did not have an important effect on the atomic concentration of the P-based species. No presence of phosphide or phosphate species was detected, and the P was deposited entirely as elemental P on Si/SiO_2_ wafers and soda lime glass as shown in Table S4, and Figure S10 and Table S5, respectively. We further analyzed the effect of the number of ALD cycles on the surface chemical composition of the RP. The elemental composition of the RP deposited on carbon paper after a different number of ALD cycles (see Table S6) revealed the presence of C, P, and O and residual content of Sn and Cl. As the number of ALD cycles grew, more RP was deposited, and the content of C dramatically decreased (as the carbon substrate gets coated). The corresponding peak fitted HR P 2p XPS spectra obtained are shown in Figure S11 together with the spectrum obtained from commercial RP powder used as a reference. The results confirmed the presence of elemental P (P–P) by the peaks of characteristic spin–orbit splitting of P 2p_3/2_ and P 2p_1/2_ at ≈129.9 and ≈130.8 eV respectively, and the lack of phosphate or any other P-based species (see Table S7) as in the case of Si/SiO_2_ and soda lime glass substrates, revealing that there was no chemical composition dependence on the type of substrate. These results indicated that the ALD process toward elemental P is not accompanied by deposition of other P-based species within the used deposition temperature range and regardless of the number of ALD cycles.

The characterization of the RP thin films was extended by spectroscopic ellipsometry measurements toward the determination of optical constants such as refractive index and extinction coefficient, UV–vis absorption coefficient, and the band gap value. The spectra of optical parameters (refractive index and extinction coefficient) of RP shown in the Figure S12 were measured in the wavelength range 200–1800 nm. All parametrizations described in the Experimental Section (TL, CL, TL+L) provided reasonable fits to ellipsometry data (Figure S12a) and yielded similar optical constants for RP ≈43 nm-thick and a surface roughness value of ≈3 nm. The thickness value was in good agreement with the thickness estimated by SEM cross sectional images (≈ 42 nm). CL and TL+L parametrizations uncovered a weak absorption tail in the NIR that could be related to the various types of defects present in the film. The obtained spectra of the refractive index and extinction coefficient of RP are shown in Figure S12b. The film was nearly transparent in the NIR, with a refractive index value of about 2.76. To the best of our knowledge, the experimentally determined optical properties (in terms of complex refractive index) of amorphous RP in the UV–vis-NIR spectral range are reported for the first time. Roth et al. estimated the refractive index value of the amorphous RP for the Li spectral line (670.78 nm) to be in the range 2.7–3.0 that fits our data (2.76).^35^ Studies in the literature related to the photocatalytic properties of RP usually present the VIS optical properties of RP by the spectral dependence of its absorption coefficient α. This can be easily calculated from the extinction coefficient *k* by the relation

α=4πk/λ

Figure S13a shows the absorption coefficient spectrum of our amorphous RP. The absorption coefficient (or the Kubelka–Munk function obtained from diffuse reflectance measurements) can be used to estimate the band gap value by constructing the Tauc plot (the dependence of (α*h*ν)^1/2^ on the photon energy *hv*). The Tauc plot corresponding to our data is presented in Figure S13b. It can be noted that the plot values are in good agreement to those reported by Kawashima studying rf sputtered amorphous RP films.^36^ The CL and TL parametrizations yielded band gap (*E*_g_) values of 1.92 and 1.86 eV, respectively. In the case of the TL+L parametrization, the Tauc plot of the absorption coefficient α, on photon energy, *E*, in the form:

1 $${\left(\alpha \cdot E\right)}^{1/2}=E_{g}-E$$

typical for an amorphous semiconductor, yielded a band gap value of 1.88 eV. All determined band gap values were close together and were consistent with literature data reported within the interval 1.74–2.4 eV confirming the semiconducting nature of the RP.^7,36,37^ Our experimentally obtained optical constants can be further used as a reference for tuning DFT calculations. The absorption coefficient values determined in this work were about 1 order of magnitude higher as compared to the corresponding DFT calculated spectra in a previous work.^37^ The Cody-Lorentz parametrization of amorphous RP electric permittivity provided (besides the band gap value) information about the transitions from the localized tail states above the valence band edge to extended states in the conduction band and/or from extended states in the valence band to localized tail states below the conduction band, the so-called Urbach edge, (up to 2.6 eV in our case).^38,39^ The Urbach energy of 327 meV characterizes the exponential spectral increase of α in this spectral region. The relation of the Urbach edge on impurity concentration and level of disorder in amorphous RP film was already discussed in an earlier work.^40^

Density functional theory (DFT) calculations were carried out to model the initial reaction steps in order to gain deeper insight into the ALD reaction mechanism behind the formation of the RP chains. Figure 5 illustrates plausible chemical reactions between TMT_3_P and SnCl_4_, while the energy profile is shown in Figure S14. A simplified SiO_2_ surface model consisting of four structural units has been used. The initial stage involves the reaction of SnCl_4_ with free hydroxyl groups on the substrate surface toward **1Sn**, which forms a stable Lewis acid–base complex **2Sn** (−91 kJ·mol^–1^) upon reacting with TMT_3_P. Subsequent release of volatile Me_3_Sn-Cl generates intermediate **3Sn**, which serves as the starting point for further reactions. For the sake of clarity, only a single Cl atom is always depicted to exchange. The second ALD cycle introducing another SnCl_4_ may proceed via two plausible pathways. The exergonic (−44 kJ·mol^–1^) pathway A involves an expected ligand exchange toward the surface intermediate **4Sn** featuring a new P–Sn bond and release of volatile Me_3_Sn-Cl. Upon further reaction with TMT_3_P and cycle repetition via the A1 route, an alternating Sn and P layers of Sn_3_P_4_ should be formed but as neither Sn_3_P_4_ nor any other tin phosphide were deposited, this reaction route is not operational. The alternative pathway A2 involves a ligand exchange accompanied by the formation of P–P bonds and release of Me_3_Sn-SnCl_3_ byproduct. When going from **4Sn** (P^3–^) to **5Sn** (P^1–^), the process involves an oxidation of the phosphorus atom along with a reduction of the original Sn^4+^ species toward Sn^3+^ in Me_3_Sn-SnCl_3_. The overall electron balance and decrease in energy (−110 kJ·mol^–1^) make this reaction pathway theoretically feasible. Subsequent reaction of **5Sn** with SnCl_4_ and repeating the cycle via pathway A2 would ultimately yield elemental phosphorus (“**4Sn**” like chain growth), with a cumulative energy balance of −242 kJ·mol^–1^ (the red pathway in Figure S14). Figure 5 further shows reaction pathway B affording the species **6Sn** upon a redox process between **3Sn** and SnCl_4_. This endergonic (+30 kJ·mol^–1^) process gives rise to Me_3_Sn-SnCl_3_ or Me_3_Sn-SnCl_2_-SnMe_3_ byproducts (Sn^3+^ or Sn^2+^) via electron transfer from the phosphorus atom (P^3–^ → P^1+^). The substitution reaction with TMT_3_P via pathway B1 affords **5Sn** (−184 kJ·mol^–1^), which reacts with SnCl_4_ and upon repetition allows a P-chain growth via a “**6Sn**” intermediate. The whole process is exergonic (−242 kJ·mol^–1^), but the chlorination step toward **6Sn** remains endergonic. An eventual parallel pathway B2 involves substitution of one chlorine atom of **6Sn** affording **7Sn**, which undergoes an elimination of another Me_3_Sn-Cl and generation of a P=P bond in **8Sn**). The overall cascade is exergonic (−136 kJ·mol^–1^), but the two endergonic steps reduce its feasibility. Hence, the A → A2 sequence via surface intermediates 3Sn → 4Sn → 5Sn seems to be the most favorable pathway toward RP.

**Figure 5:**
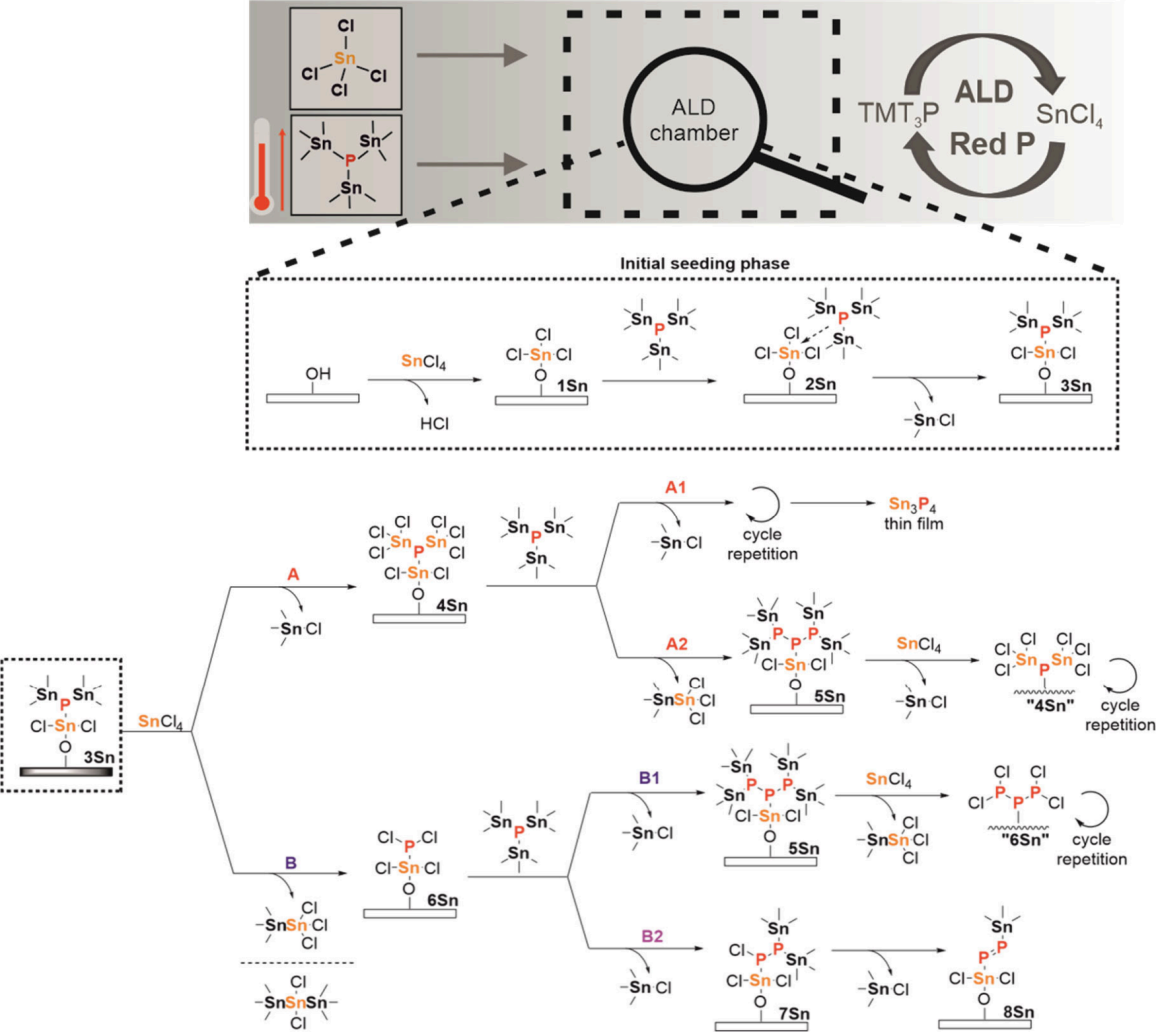
Schematic diagram of the ALD process and plausible reaction pathways between TMT_3_P and SnCl_4_.

In summary, a new synthetic route for RP is presented by means of ALD extending the portfolio of ALD precursors and filling the gap in missing deposition of elemental P. The RP growth was demonstrated on Si/SiO_2_ wafers, soda lime glass, and carbon paper, by combining SnCl_4_ and home-synthesized TMT_3_P within a temperature deposition range between 150 and 250 °C. The resulting RP thin films were characterized by different techniques, revealing the phosphorus was deposited entirely as elemental RP without any other phosphorus species, and an optical bandgap of ≈1.9 eV confirming RP’s semiconducting nature. Based on DFT calculations, a plausible ALD reaction mechanism combining ligand exchange and redox steps has been proposed.

## Supplementary Material



## Notes

Description of the experimental methods including the chemical treatment of carbon paper, cleaning of Si/SiO_2_ wafers and soda lime glass substrates, atomic layer deposition of RP and characterization techniques. Supplementary Figures S1: Optical image of soda lime glass substrates coated with 650 ALD cycles at different deposition temperatures. S2: Optical image of soda lime glass substrates coated with different number of ALD cycles at 200 °C. Figures S3–S13. S3: Cross-sectional SEM images of RP thin films. S4: Top-view SEM images of RP thin films deposited on Si/SiO_2_ wafers. S5: AFM images from RP thin films deposited on Si/SiO_2_ wafers. S6: XRD pattern obtained from a RP thin film. S7: Top-view SEM image obtained from RP thin film deposited on soda lime glass. S8: XPS survey spectrum obtained from RP thin films deposited on Si/SiO_2_ wafers. S9: XPS survey spectrum obtained from RP thin films deposited on soda lime glass. S10: High-resolution P 2p XPS spectrum from RP deposited on soda lime glass. S11: High-resolution P 2p XPS spectra from red P powder and RP deposited on carbon paper. S12: The fit of ellipsometry parameters along with the refractive index and extinction coefficient of RP determined from ellipsometry. S13: Calculated energies of reaction pathways of TMT_3_P and SnCl_4_. Supplementary Tables S1–S7. Table S1: Mean roughness value obtained from Si/SiO_2_ wafers after different number of RP ALD cycles. Table S2: XPS elemental composition of elemental RP thin films deposited on Si/SiO_2_ wafers. Table S3: XPS elemental composition of elemental RP thin films deposited on soda lime. Table S4: Atomic concentration of the different P-based species obtained at different deposition temperatures. Table S5: Atomic concentration of the different P-based species on soda lime glass. Table S6: XPS elemental composition of elemental RP deposited on carbon paper. Table S7: Atomic concentration of the different P-based species deposited on carbon paper. Density functional theory calculation details including the coordinates (PDF)
