# Supplementary material for: Red Phosphorus by Atomic Layer Deposition
Source: Nano Lett. 2026 Apr 28;26(18):6161–7. doi: 10.1021/acs.nanolett.6c01078 (PMC13178126; doi:10.1021/acs.nanolett.6c01078)
Supplement: Supplementary file 1 [file nl6c01078_si_001.pdf]

Supporting Information

Red Phosphorus by Atomic Layer Deposition

Raul Zazpe,[a,b], Jaroslav Charvot,[c] Jhonatan Rodriguez-Pereira,[a,b] Jiří Váňa,[c] Luděk Hromádko,[a,b] David Pavliňák,[b] Jan Mistrik,[a,d] Michal Kurka,[a] Jan M. Macak[a,b], and Filip Bureš*[c]*

[a]Center of Materials and Nanotechnologies, Faculty of Chemical Technology
University of Pardubice
Studentska 95, 532 10 Pardubice, Czech Republic
E-mail: raul.zazpe@upce.cz

[b]Central European Institute of Technology
Brno University of Technology
Purkynova 123, 612 00 Brno, Czech Republic

[c]Institute of Organic Chemistry and Technology, Faculty of Chemical Technology
University of Pardubice
Studentska 95, 532 10 Pardubice, Czech Republic
E-mail: filip.bures@upce.cz

[d]Institute of Applied Physics and Mathematics, Faculty of Chemical-technology
University of Pardubice
Studentska 95, 532 10 Pardubice, Czech Republic

Experimental section

Chemical treatment of carbon paper (CP) substrate

Commercial CP (CT GDS310) (Fuel cell store) were chemically treated before ALD processes due to the chemical inertness of the CP (i.e. lack of chemical active sites on the surface). The chemical treatment was carried out using HNO_3:H_2SO_4 (1:3) acidic solution under sonication for 1 hour at 60 °C to generate oxygen-based active nucleation sites on the surface to promote the growth of RP. Following, the treated CP were thoroughly rinsed with distilled water until rinse water is neutral. Finally, the CP was dried in the oven at 80 °C overnight.

Cleaning of Si/SiO_2 wafers and soda lime glass substrates

Before ALD processes the Si/SiO_2 (native) wafers and soda lime glass substrates were subjected to a multi-step process to remove potential contaminants (particles, grease) and improve film adhesion and uniformity. The substrates were dipped in different solvent baths, namely, acetone, isopropanol and distilled water under sonication for 5 minutes at each solvent. Next, they were dried using N_2 blow.

Atomic layer deposition of RP

The synthesis of RP was carried out by thermal ALD (Beneq-TFS200) within a temperature range from 150 to 250°C using in-house synthesized TMT_3P and tin tetrachloride ($SnCl_4$, Merck, 99.995% trace metals basis). The synthesis of TMT_3P was described in a previous published work.[1] The TMT_3P was heated up to increase the vapor pressure to 90 °C while $SnCl_4$ was kept at room temperature (20 °C). A flow rate of 500 standard cubic centimeters per minute (sccm) of N_2 (99.9999%) was applied as inert carrier gas in a continuous flow mode with a base pressure value of around 2 mbar. One ALD cycle was composed of the next sequence: TMT_3P pulse (500 ms) – N_2 purge (10 s) – $SnCl_4$ dose (75 ms) – N_2 purge (10 s).

Characterization techniques

The morphology and the thickness of the RP deposited on carbon papers, Si/SiO_2 wafers and soda lime glass were evaluated using a field-emission scanning electron microscope (FE-SEM) JEOL JSM 7500F. SEM characterization was carried out applying an accelerating voltage of 5 kV, a beam current of 20 mA and a probe current of 30 pA. r-Filter was applied to combine signals (1 : 1) from secondary and backscattered electrons. The thickness of the RP thin films was measured by cross-sectional SEM images and statistically evaluated using proprietary Nanomeasure software.

X-ray diffraction (XRD) analysis was performed using Panalytical Empyrean with a Cu tube and a Pixcel3D detector. Grazing incidence XRD was performed to obtain the diffraction spectra of the RP thin films. The incident angle was 1 degree. The patterns were recorded in the range of 5–65°, the step size was 0.026 degrees, and the time per step was 11 s.

The roughness and morphology of the RP thin films were determined by atomic force microscopy (AFM) in air using the NTEGRA (NT-MDT) system and applying tapping mode with a HA-HR tip (ScenSans) and a step of 8 nm. The roughness value was obtained as the mean value of 3 measurements of a scanned area of 5 × 5 μm^2.

X-ray photoelectron spectroscopy (XPS) (ESCA2SR, Scienta-Omicron) was employed to evaluate the surface chemical composition of deposited RP using a monochromatic AlKα (1486.7 eV) X-ray source operated at 200 W. The binding energy scale correction was carried out using the adventitious carbon C 1s at 284.8 eV. The survey and high-resolution (HR) spectra were recorded at a pass energy of 150 and 30 eV, respectively. Additionally, a commercial RP powder (Sigma-Aldrich, > 99.99%) was measured using a CN-10 charge neutralizer operated at 5 µA and 1.5 eV, since it was the only sample requiring charge neutralization. Data analysis was performed with the CasaXPS program (Casasoftware Ltd). Quantitative analysis was performed using the elemental sensitivity factors provided by the manufacturer.

Raman spectroscopy was carried out by equipment WiTec confocal Raman system Alpha 300R by 532 and 633 nm laser. Due to the intense vibration of the silicon crystal lattice in the region of expected phosphorus vibrations, the samples were analyzed only on a glass substrate. To prevent thermal damage to the sample due to the laser dose, a set of tests was performed with different acquisition times and power settings. The most effective setting ultimately turned out to be a 532 nm laser with a power of 20 mW, a 50x objective, and a short acquisition time with a measurement accumulation of 20x0.1s.

Spectra of ellipsometric parameters were recorded using the VASE ellipsometer (Woollam Co. Ltd) over the range of 0.7-6.0 eV (207-1770 nm) at three incidence angles: 50°, 60°, and 70°. The WVASE32 software was used to fit the data. The sample model consisted of a naturally oxidised c-Si substrate covered by a homogeneous RP film, with surface roughness approximated by Bruggemann's effective medium (50% voids). Optical constants for c-Si and SiO_2 were taken from the software database. The dielectric function of red phosphorus was modelled by three different parameterizations: Tauc-Lorentz (TL), Cody-Lorentz (CL) and summation of Tauc-Lorentz and Lorentz (TL+L) oscillators. The band gap value was determined either directly from oscillator formula (TL, CL) or from the Tauc plot (TL+L).

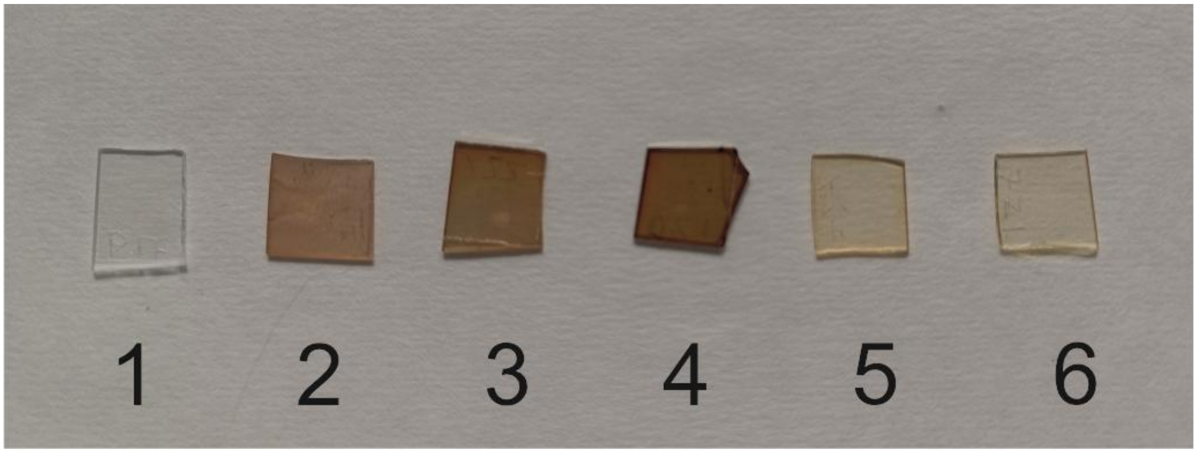

Figure S1. Optical picture of soda lime glass substrates, coated with RP after 650 ALD cycles at different deposition temperatures: (1) Blank, (2) 150 °C, (3) 175 °C, (4) 200 °C, (5) 225 °C and (6) 250°C.

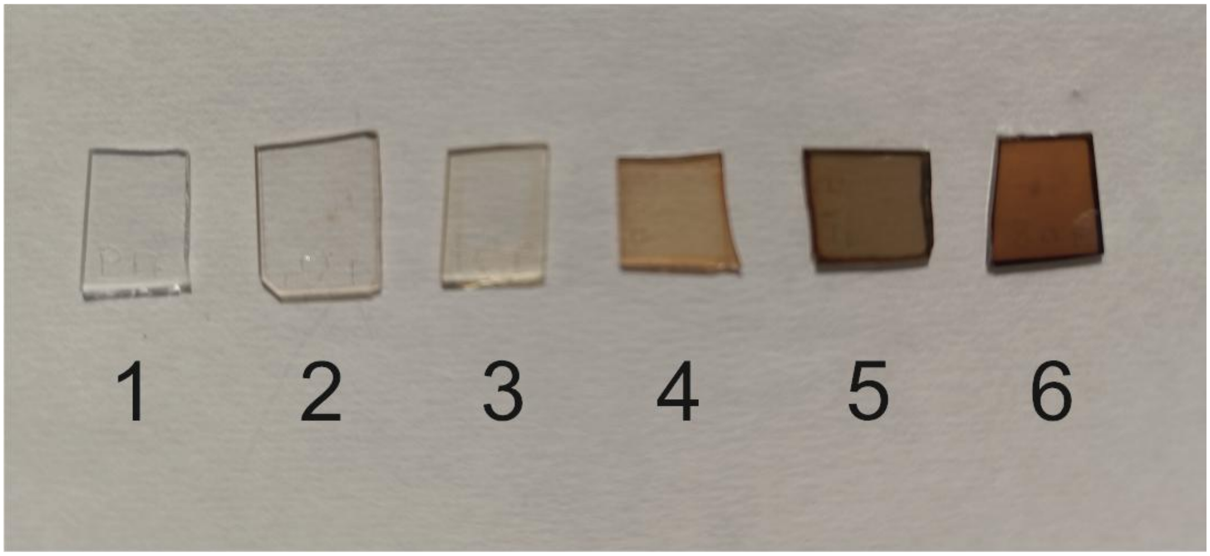

Figure S2. Optical picture of soda lime glass substrates, coated with RP after different number of ALD cycles of at 200°C: (1) Blank, (2) 25, (3) 150, (4) 500, (5) 750 and (6) 1000.

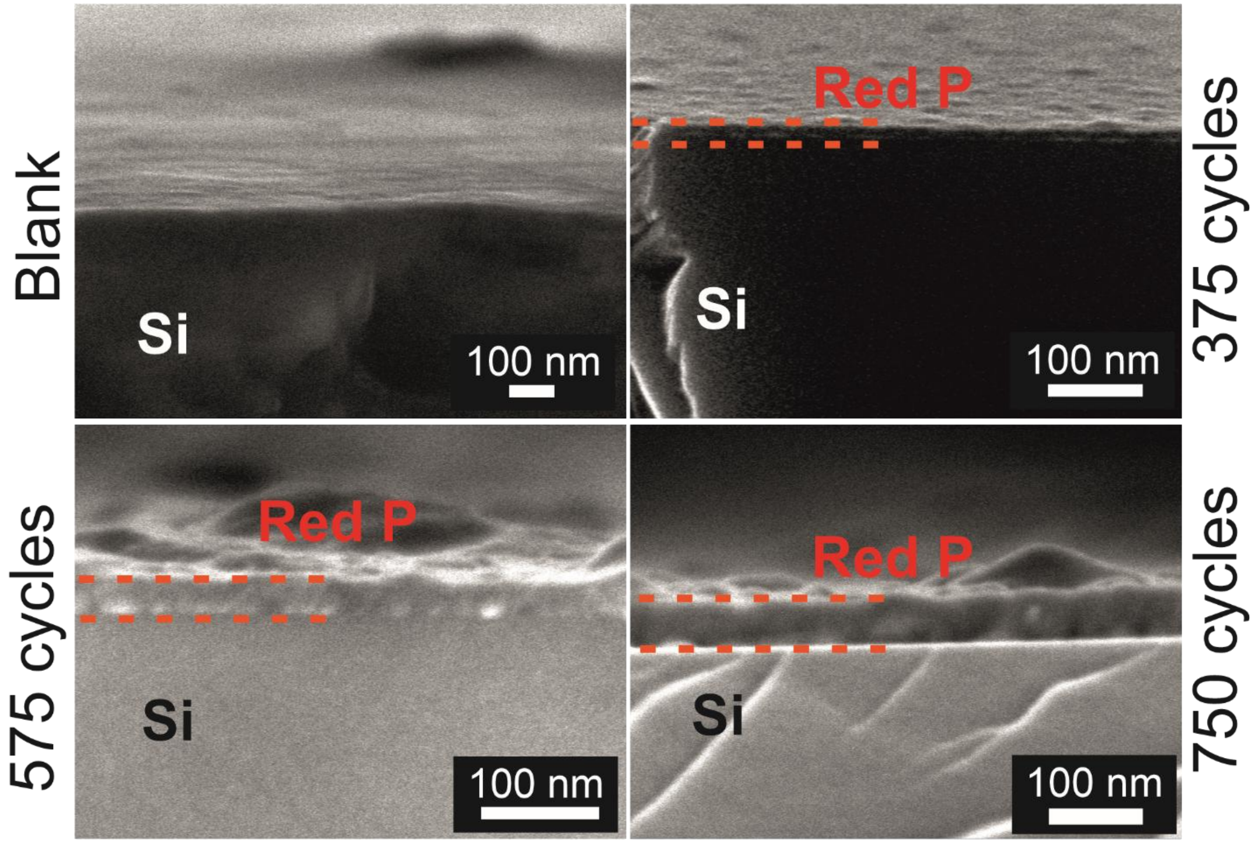

Figure S3. Cross-sectional SEM images of RP thin films deposited on Si/SiO_2 wafers at 200 °C after different number of ALD cycles: 0 (blank), 375, 575 and 750.

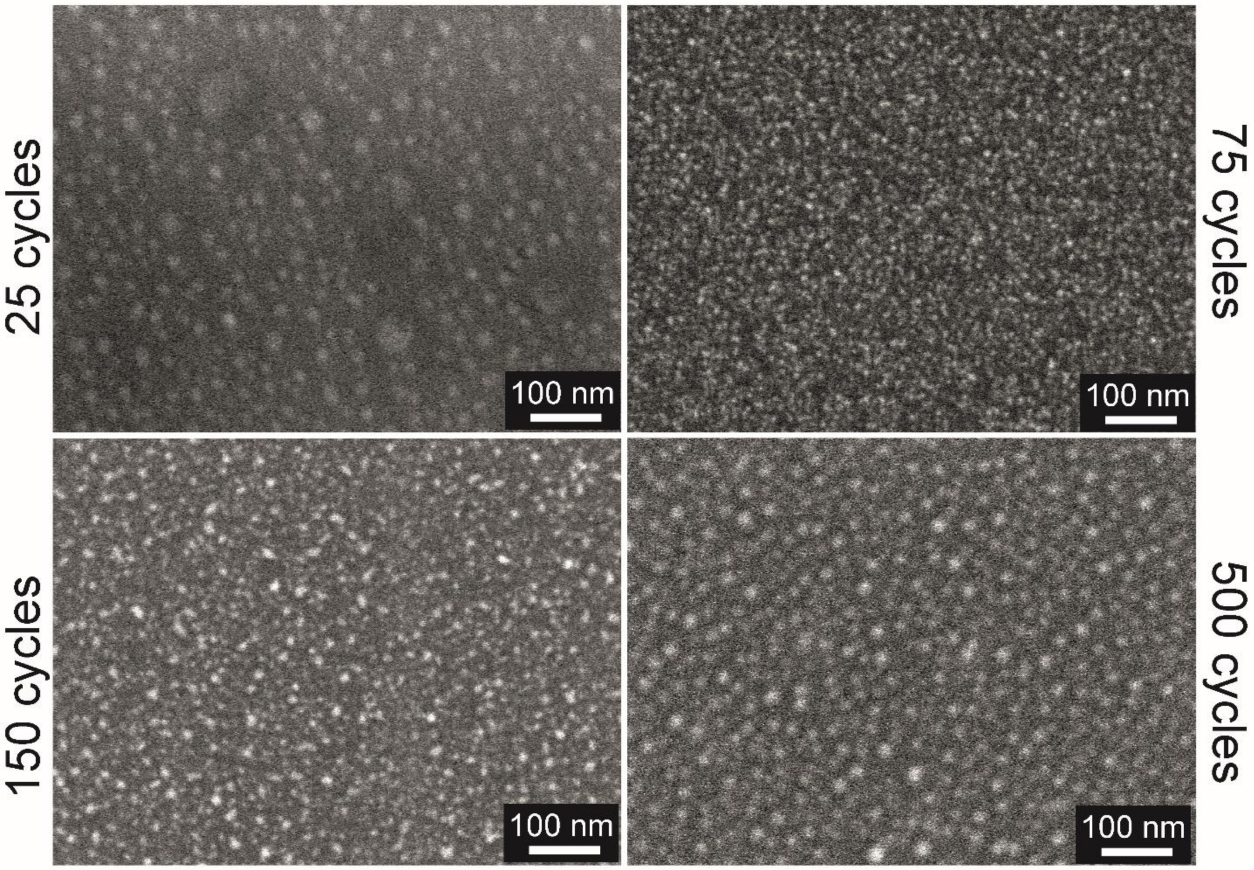

Figure S4. Top-view SEM images of RP thin films deposited on Si/SiO_2 wafers at 200 °C after different number of ALD cycles: 25, 75, 150 and 500.

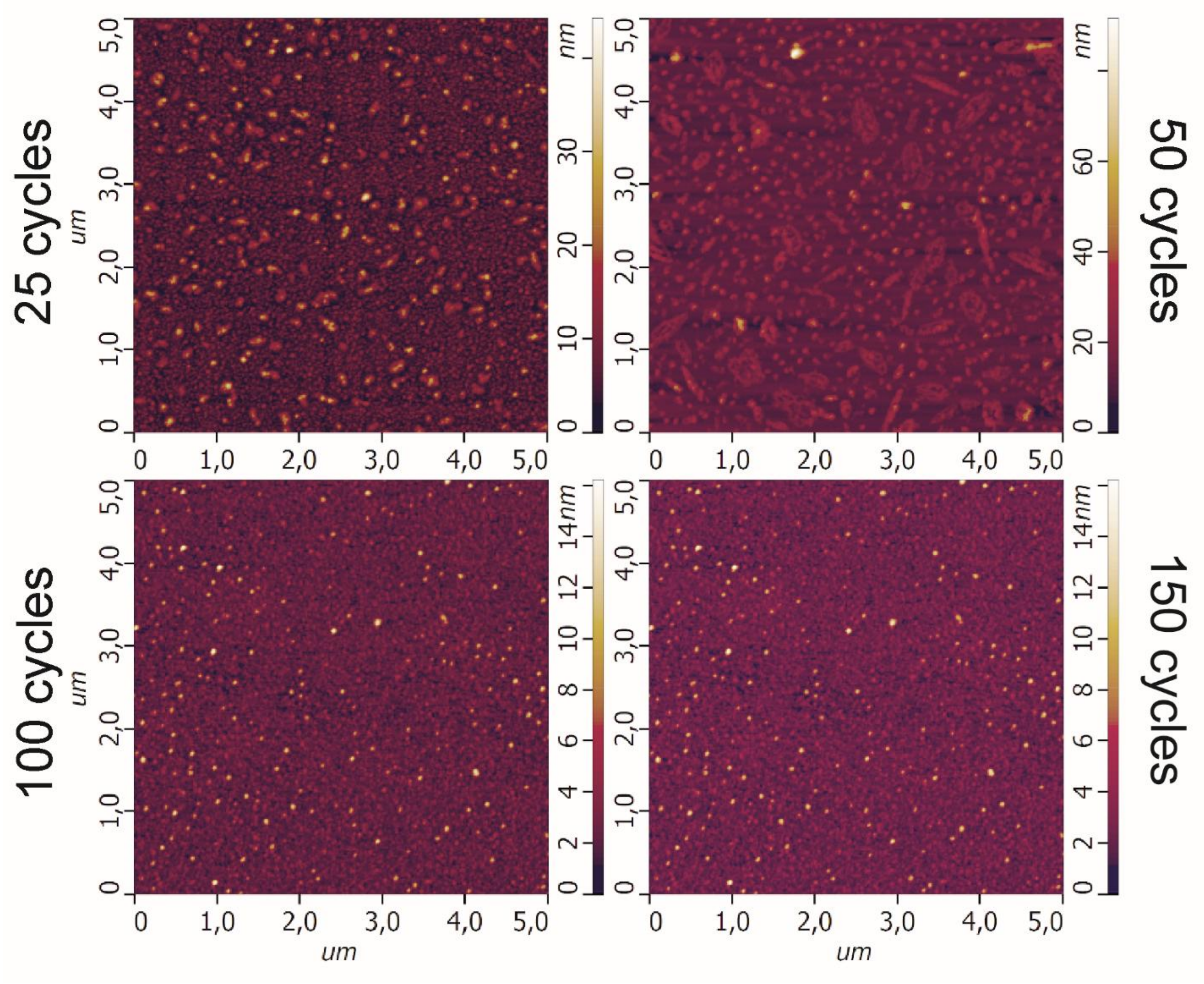

Figure S5. AFM images from RP thin films deposited on Si/SiO_2 wafers at 200 °C after different number of ALD cycles.

Table S1. Mean roughness value obtained from Si/SiO_2 wafers after different number of RP ALD cycles at 200 °C.

# ALD cycles	Roughness [nm]
25	4.1 ± 0.2
50	5.8 ± 1.4
100	4.2 ± 2.4
150	6.4 ± 0.6

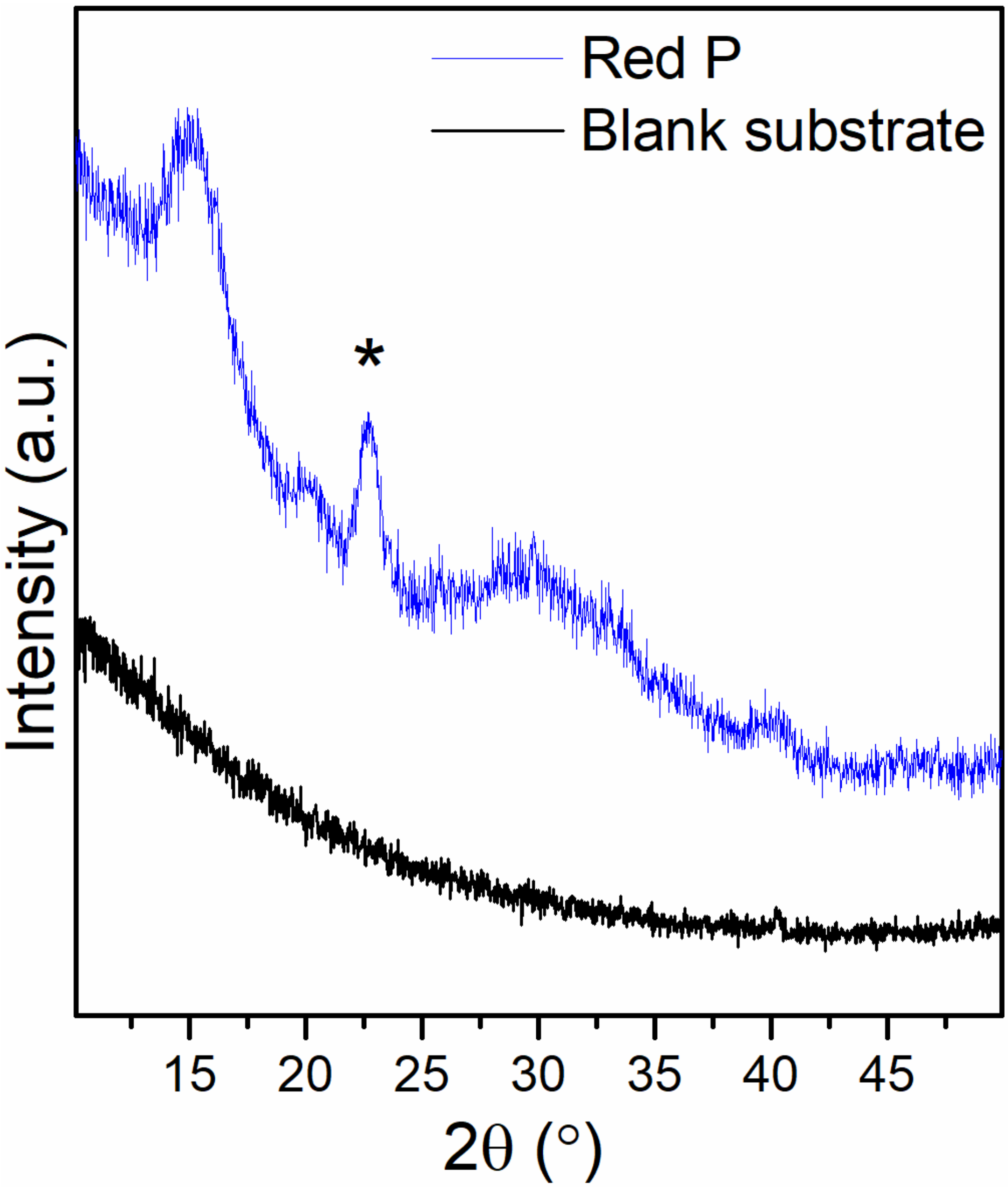

Figure S6. XRD pattern obtained from a RP thin film ≈ 30 nm-thick deposited on Si/SiO_2 wafer at 200 °C.

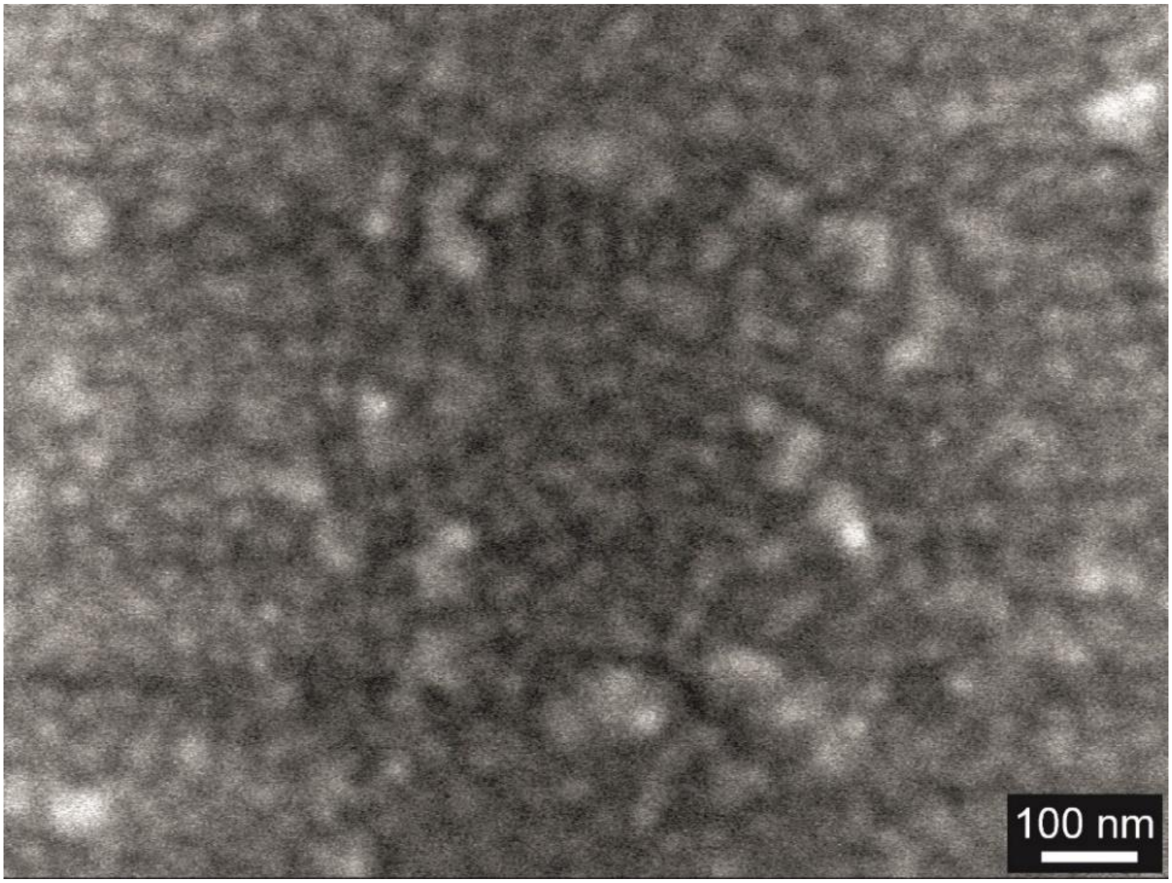

Figure S7. Top-view SEM image obtained from RP thin film deposited on soda lime glass at 200 °C after 750 cycles.

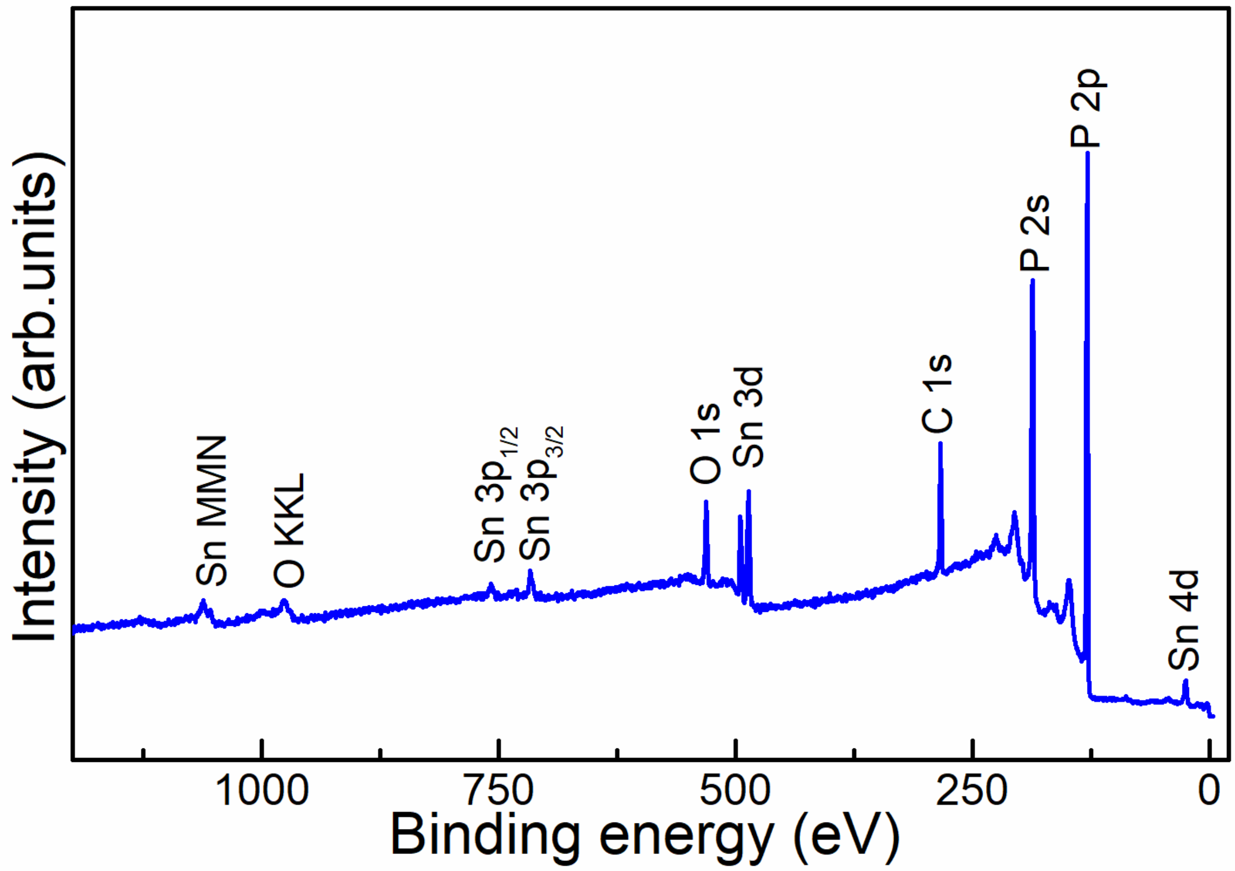

Figure S8. XPS survey spectrum obtained from RP thin film deposited on Si/SiO_2 wafers at 200°C after 650 ALD cycles applying 500 ms TMT_3P dose and 75 ms $SnCl_4$ dose.

Table S2. XPS elemental composition of elemental RP thin films deposited after 650 ALD cycles on Si/SiO_2 wafers at different temperatures (150, 175, 200, 225 and 250 °C) applying 500 ms TMT_3P dose and 75 ms $SnCl_4$ dose.

**Deposition temperature**	**Atomic concentration [%]**					
	**C**	**O**	**Sn**	**P**	**Cl**	**Si**
**150 °C**	21.3	5.6	8.8	55.9	8.2	-
**175 °C**	16.6	5.1	5.2	70.7	2.2	-
**200 °C**	23.7	9.3	2.3	64.7	-	-
**225 °C**	14.5	4.7	1.3	69.4	-	9.8
**250 °C**	12.8	10.4	1.6	51.6	-	23.3

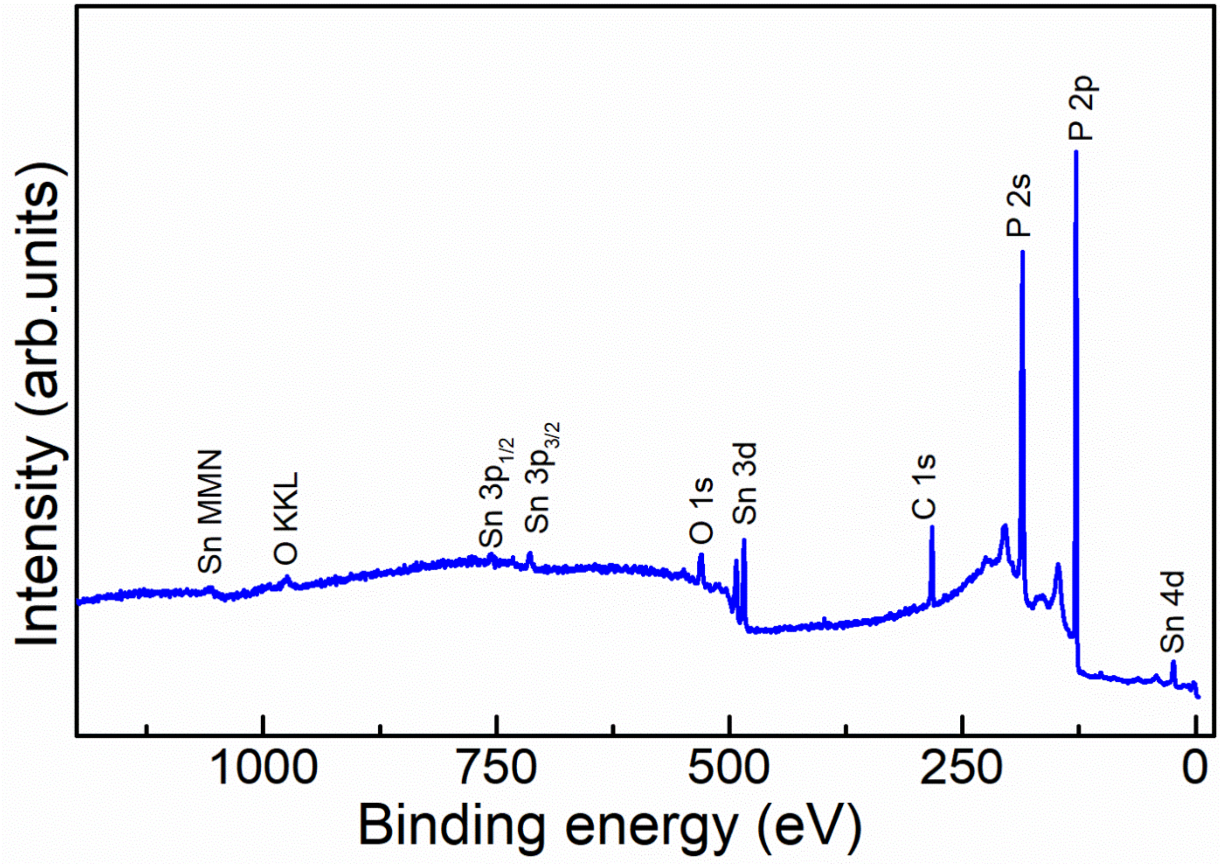

Figure S9. XPS survey spectrum obtained from RP thin film deposited on soda lime glass at 200°C after 750 ALD cycles applying 500 ms TMT_3P dose and 75 ms $SnCl_4$ dose.

Table S3. XPS elemental composition of elemental RP thin film deposited on soda lime glass at 200°C after 750 cycles applying 500 ms TMT_3P dose and 75 ms $SnCl_4$ dose.

Substrate	Atomic concentration [%]					
	C	O	Sn	P	Cl	Si
**Soda lime glass**	16.1	4.8	1.8	76.3	-	1.00

Table S4. Atomic concentration of the different P-based species from the high-resolution peak fitted XPS P 2p spectra obtained from RP thin films deposited at different temperatures (150, 175, 200, 225 and 250 °C) applying 500 ms TMT_3P dose and 75 ms $SnCl_4$ dose.

**Deposition temperature (°C)**	**P Atomic concentration [%]**		
	**P$^-$**	**P-P**	**P**-O
**150**	-	100	-
**175**	-	100	-
**200**	-	100	-
**225**	-	100	-
**250**	-	100	-

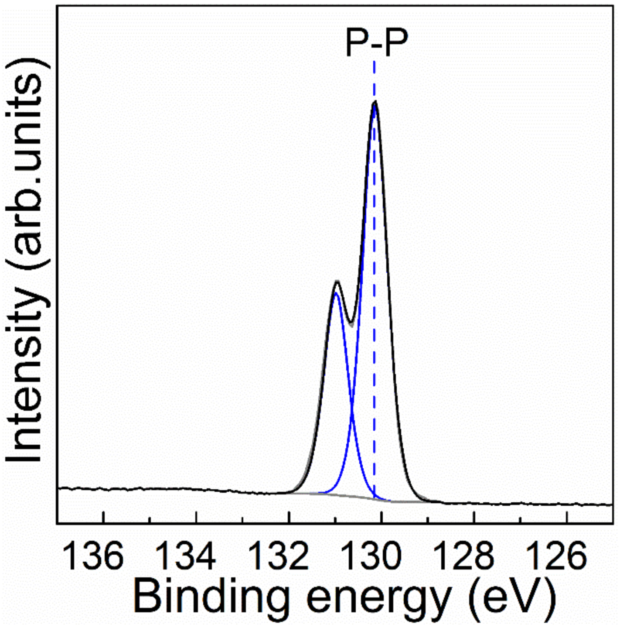

Figure S10. High-resolution P 2p XPS spectrum from RP deposited on soda lime glass at 200°C after 750 ALD cycles applying 500 ms TMT_3P dose and 75 ms $SnCl_4$ dose.

Table S5. Atomic concentration of the different P-based species from the high-resolution peak fitted XPS P 2p spectra, obtained from RP thin films, deposited on soda lime glass at 200 °C after 750 cycles.

**Substrate**	**P Atomic concentration [%]**		
	**P$^-$**	**P-P**	**P**-O
**Soda lime glass**	-	100	-

Table S6. XPS elemental composition of elemental RP deposited at 200 °C on carbon paper after different number of ALD cycles and applying 500 ms TMT_3P dose and 75 ms $SnCl_4$ dose.

# ALD cycles	Atomic concentration [%]				
	C	O	Sn	P	Cl
**25**	79.6	8.2	1.8	9.6	0.8
**75**	54.4	8.9	3.4	32.2	1.1
**150**	34.5	4.9	1.3	59.3	-

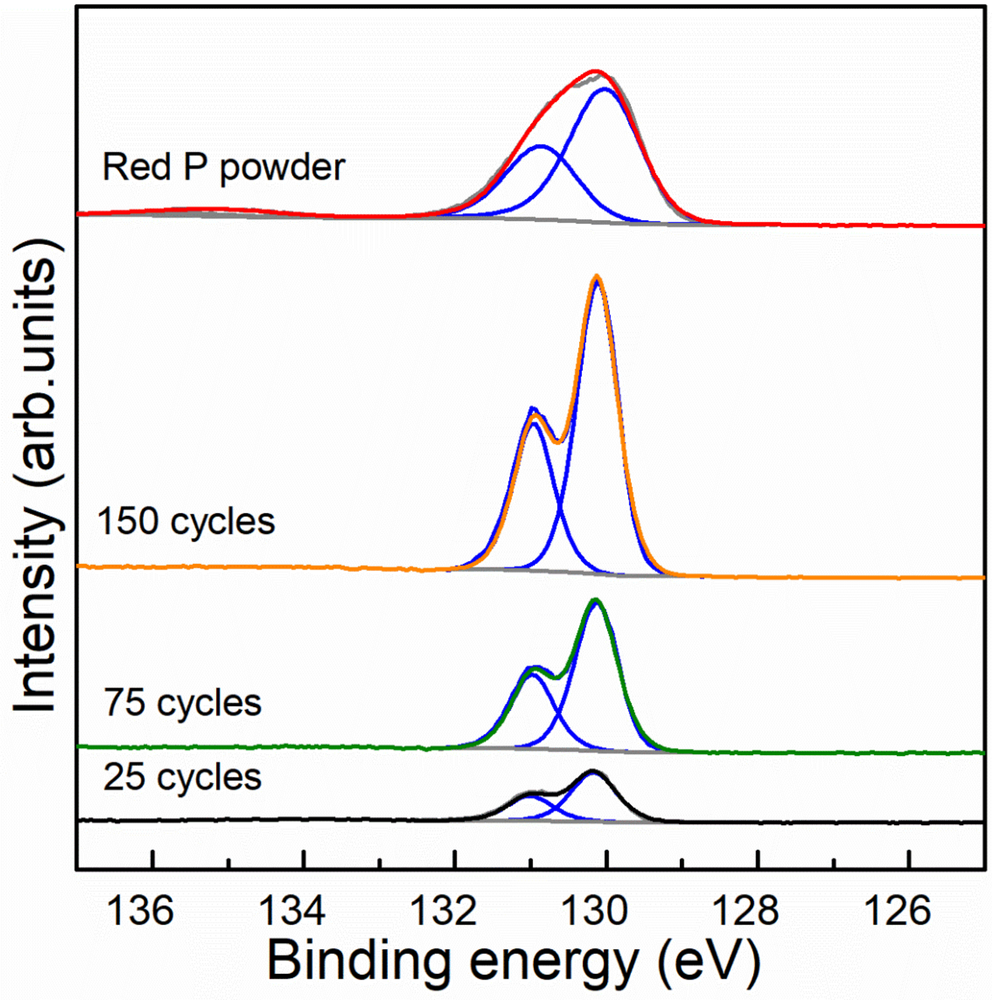

Figure S11. High-resolution P 2p XPS spectra from red P powder (reference) and RP deposited on carbon paper at 200 °C after different number ALD cycles: 25, 75 and 150, applying 500 ms TMT_3P dose and 75 ms $SnCl_4$ dose.

Table S7. Atomic concentration of the different P-based species from the high-resolution peak fitted XPS P 2p spectra obtained from RP thin films, deposited on carbon paper at 200 °C after different number of ALD cycles (25, 75 and 150) applying 500 ms TMT_3P dose and 75 ms $SnCl_4$ dose.

# ALD cycles	P Atomic concentration [%]		
	P^-	P-P	P-O
**25**	-	100	-
**75**	-	100	-
**150**	-	100	-

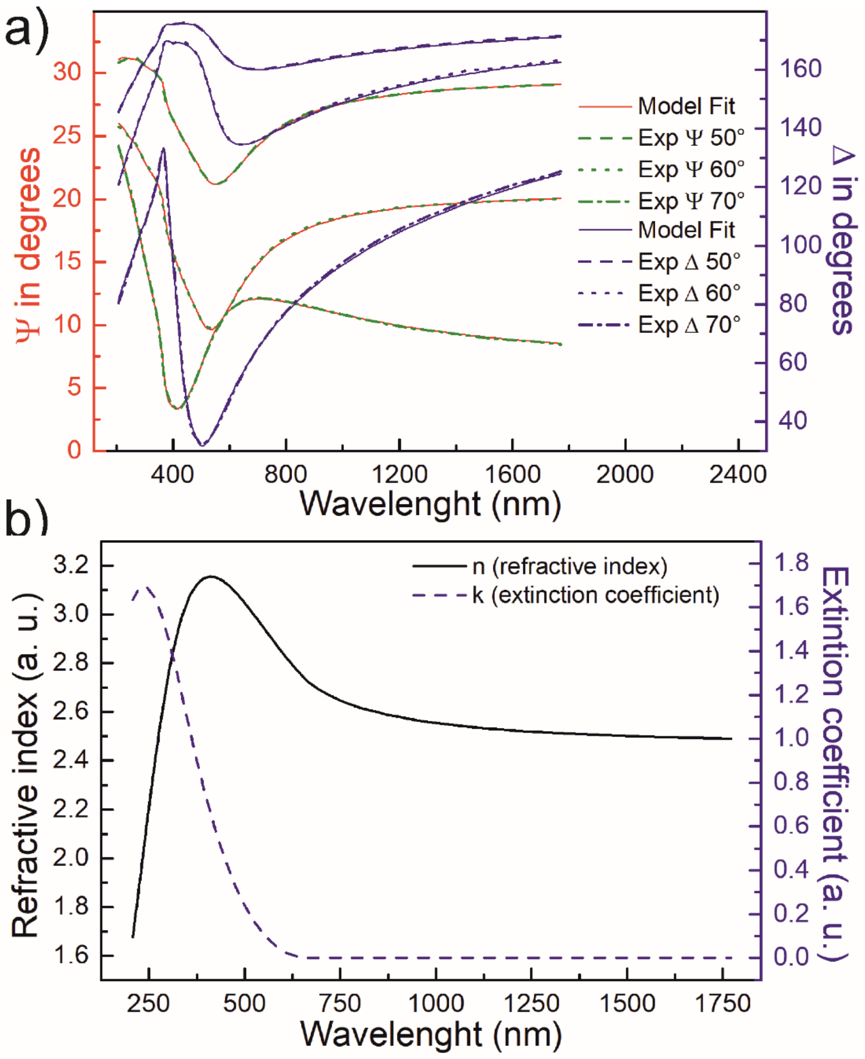

Figure S12. (a) The fit of ellipsometry parameters Ψ and Δ for the incidence angles of 50°, 60°, and 70°. (b) The refractive index and extinction coefficient of RP determined from ellipsometry (Cody-Lorentz parameterization).

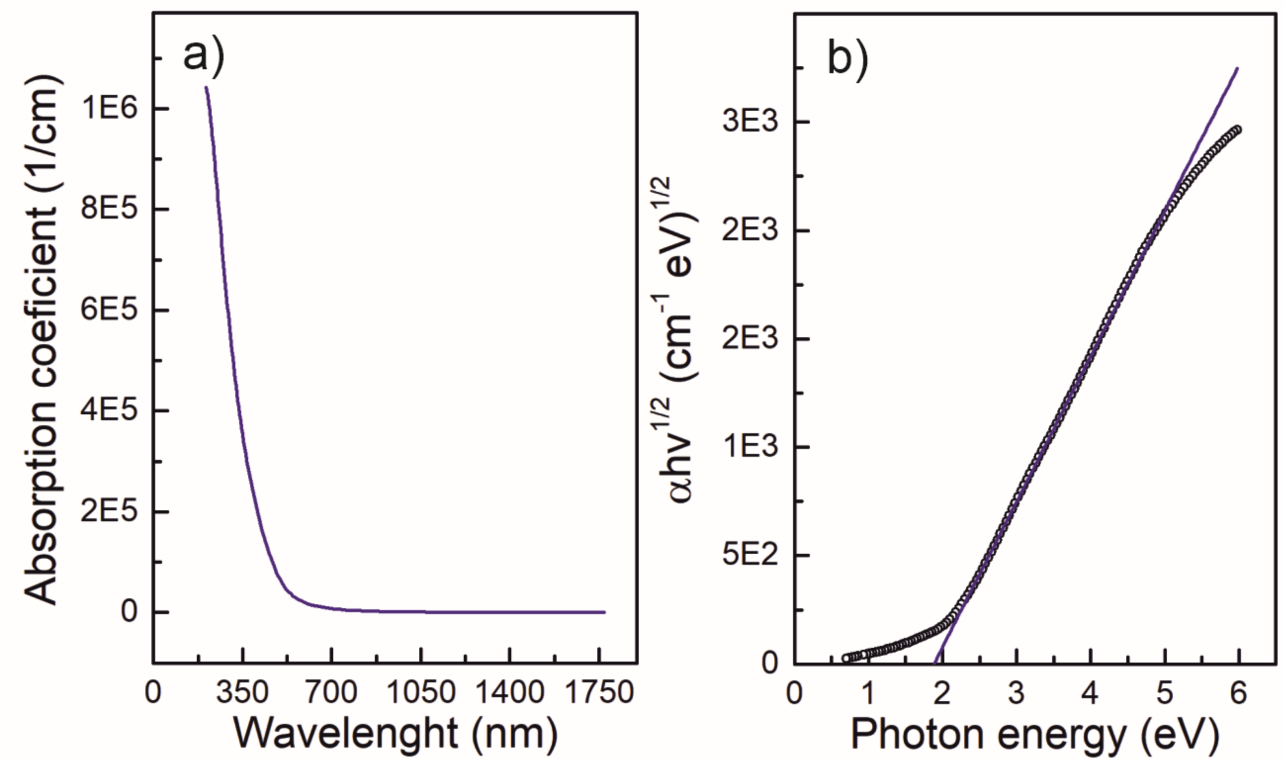

Figure S13. (a) The absorption coefficient of the amorphous RP film calculated from extinction coefficient derived from ellipsometry, and (b) the corresponding Tauc plot.

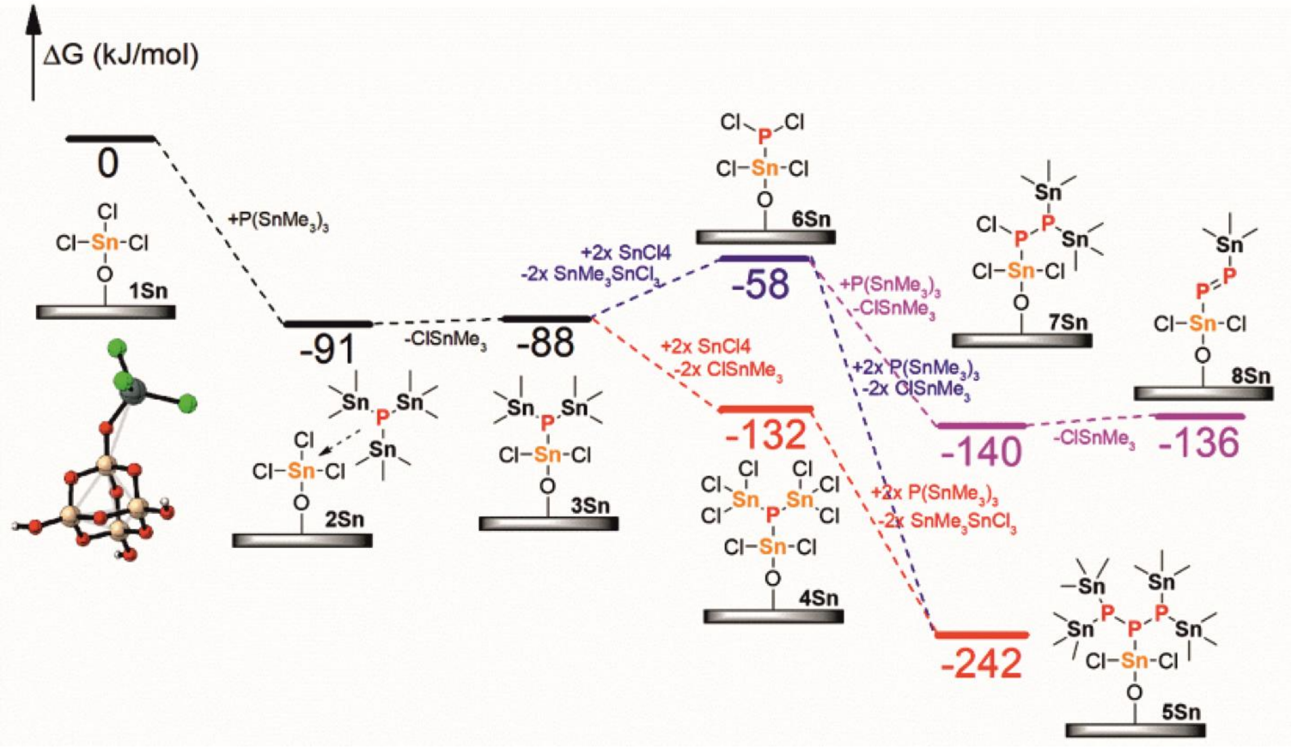

Figure S14. Calculated energies of reaction pathways of TMT_3P and $SnCl_4$.

Density Functional Theory (DFT)

All calculations were performed using Gaussian 16[2] software package using the B3LYP[3,4] functional with def2TZVP[5] basis set and gd3bj empirical dispersion correction.[6] All reported minima were confirmed by the calculation and diagonalization of their Hessian matrices. The reported energies are Gibbs free energies at 298.15 K and the 1 bar standard state.

The following formal reactions were balanced for the construction of energy plots:

1 + $P(SiMe_3)_3$ → **3** + $ClSiMe_3$

3 + 2 $SnCl_4$ → **4** + 2 $ClSiMe_3$

4 + 2 $P(SiMe_3)_3$ → **5** + 2 $SiMe_3SnCl_3$

3 + 2 $SnCl_4$ → **6** + 2 $SiMe_3SnCl_3$

6 + $P(SiMe_3)_3$ → **7** + $ClSiMe_3$

7 → **8** + $ClSiMe_3$

Coordinates

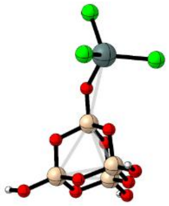

21
1 0 1 scf done: -3508.601352 Sum of electronic and thermal Free Energies -3508.570542

Si	-0.6858260	-1.6553470	-1.4417260
Si	-3.1550640	-1.6305440	-0.1265220
Si	-1.4691130	-3.8756330	0.0751610
Si	-0.7828970	-1.4899580	1.3665140
O	-0.0417280	-1.0445670	-0.0451280
O	-2.2663690	-1.1965840	-1.4530230
O	-0.6906660	-3.2912860	-1.2630500
O	-3.0041740	-3.2727630	-0.0335550
O	-2.3567310	-1.0248330	1.1771280
O	-0.7801690	-3.1330080	1.3696060
O	-1.3882190	-5.4730610	0.2382780

H	-1.6771160	-6.0442690	-0.4768910
O	-0.0779880	-0.9200620	2.6934850
H	-0.1189840	0.0179040	2.8935370
O	-4.6723890	-1.0974090	-0.1483950
H	-5.2545190	-1.3356810	-0.8734050
O	0.1468170	-1.1432600	-2.7173360
Sn	1.7103720	0.0205050	-2.7246060
Cl	2.2759850	0.2827800	-4.9406900
Cl	3.4236850	-0.9914500	-1.5643600
Cl	1.1481000	2.0542910	-1.7941660

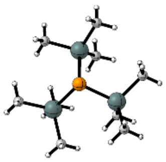

40

$P(SnMe_3)_3$ 0 1 scf done: -1344.020190 Sum of electronic and thermal Free Energies -1343.757309

P	0.0008140	-0.0003680	-1.3080000
C	1.3883220	-3.5057680	-1.1843290
H	1.3982650	-4.4996580	-0.7368330
H	1.0810680	-3.5846090	-2.2260780
H	2.3927230	-3.0867360	-1.1426750
C	0.6405830	-2.1380430	1.9645320
H	0.5948050	-3.1409650	2.3896940
H	1.6631170	-1.7723700	2.0401480
H	-0.0161240	-1.4811140	2.5305360
C	-1.9883770	-3.0351090	-0.2109360
H	-2.6639330	-2.4533630	0.4146000
H	-2.3456950	-3.0065820	-1.2393680
H	-1.9868560	-4.0679120	0.1372400
C	3.6266260	-0.1976830	-0.2094230
H	3.7846880	-0.5190640	-1.2379670
H	4.5174760	0.3233460	0.1411510
H	3.4641690	-1.0755800	0.4143770
C	1.5312630	1.6202290	1.9681270
H	1.2953990	0.7210200	2.5329300
H	2.4222330	2.0843950	2.3917550
H	0.7009760	2.3200030	2.0466750
C	2.3350370	2.9587020	-1.1787530
H	2.5548520	2.7348150	-2.2215560
H	1.4695390	3.6182030	-1.1335780
H	3.1915120	3.4636620	-0.7319840
C	-2.1702160	0.5160620	1.9667620
H	-3.0223340	1.0510630	2.3869500
H	-2.3543660	-0.5544710	2.0458280
H	-1.2771640	0.7676400	2.5349110
C	-3.7278560	0.5424770	-1.1828650

H	-3.8654040	-0.5369870	-1.1386910
H	-4.5942520	1.0306730	-0.7365340
H	-3.6430910	0.8454610	-2.2253910
C	-1.6367030	3.2382290	-0.2123810
H	-0.7982970	3.5350540	0.4161950
H	-1.4294600	3.5329730	-1.2401950
H	-2.5342030	3.7521870	0.1315430
Sn	1.9114170	1.1246180	-0.1097070
Sn	0.0170150	-2.2187930	-0.1122930
Sn	-1.9291920	1.0925940	-0.1110750

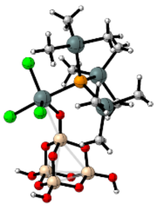

61

2Sn 0 1 scf done: -4852.680517 Sum of electronic and thermal Free Energies -4852.362338

Si	-2.6054130	0.2447050	-0.4473930
Si	-4.5447200	0.2261500	1.5977440
Si	-5.3183560	0.6757400	-1.0648370
Si	-4.4696260	-1.8625020	-0.2621430
O	-2.9437290	-1.3751780	-0.6254890
O	-3.0109930	0.5847500	1.1217670
O	-3.7357950	1.0039410	-1.3808890
O	-5.5305870	1.0190090	0.5370300
O	-4.7557230	-1.3789860	1.2908520
O	-5.4863050	-0.9582740	-1.1962550
O	-6.3553790	1.4232010	-2.0450510
H	-6.2682510	2.3698110	-2.1805450
O	-4.6029070	-3.4533420	-0.4878070
H	-5.4545910	-3.8754200	-0.3528410
O	-4.8387430	0.5439010	3.1495520
H	-4.5749270	1.3966670	3.5042260
O	-1.0848100	0.5598570	-0.7836460
Sn	0.2101560	1.8598000	0.0866510
Cl	-1.4152400	3.5891280	0.0269180
Cl	1.7733650	3.0880130	-1.2648840
P	1.7866390	-0.1844080	-0.0196260
C	-0.8153670	-1.8886850	2.1522020
H	-1.0437180	-2.5806730	2.9620310
H	-1.1992580	-0.9019490	2.3982950
H	-1.2832320	-2.2377020	1.2350820
C	2.1857390	-3.6543520	1.2429700
H	2.1212510	-4.3844800	2.0494770
H	1.6558990	-4.0407480	0.3751040
H	3.2328180	-3.5014860	0.9876240

C	2.3800590	-1.0452110	3.6168090
H	3.4470570	-0.9980460	3.4071710
H	2.0117590	-0.0536030	3.8659640
H	2.2141780	-1.7193070	4.4568200
C	-0.1560780	-2.8438860	-1.9527830
H	-0.4153780	-3.2657520	-2.9237850
H	0.2345310	-3.6371630	-1.3188790
H	-1.0454270	-2.4125380	-1.5019510
C	3.2451420	-2.2316330	-2.7426280
H	3.5459690	-2.9107220	-1.9460590
H	3.1522310	-2.7965300	-3.6699860
H	4.0075190	-1.4656580	-2.8711290
C	0.7748600	0.2118410	-3.6367120
H	-0.2235270	0.5576760	-3.3797460
H	1.4699700	1.0460180	-3.5730320
H	0.7790470	-0.1930420	-4.6481060
Cl	0.4512800	1.9454750	2.4709110
C	5.3099710	-1.1725970	0.6944400
H	6.3680880	-0.9222950	0.7693620
H	4.9674540	-1.5439140	1.6581890
H	5.1841270	-1.9508660	-0.0564470
C	4.2570120	2.1276880	1.6089770
H	3.7684640	1.7748180	2.5143830
H	5.2930550	2.3847990	1.8269110
H	3.7312400	3.0038070	1.2367110
C	4.8106480	1.2274570	-1.8596890
H	5.6021510	0.5769080	-2.2287660
H	3.9609050	1.1971390	-2.5367290
H	5.1728550	2.2523060	-1.8060110
Sn	4.1960550	0.5890130	0.1075650
Sn	1.3400980	-1.3271360	-2.2456620
Sn	1.3130860	-1.7836460	1.8948810

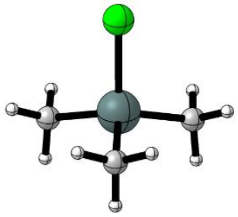

14

ClSnMe₃ 0 1 scf done: -794.472244 Sum of electronic and thermal Free Energies -794.400613

C	-0.9323160	-0.1280930	-2.0751510
H	-2.0178010	-0.1340090	-2.1733460
H	-0.5259030	0.7280210	-2.6105080
H	-0.5261710	-1.0437180	-2.5010440
C	-0.9302980	-1.7332920	1.1490780
H	-0.5230460	-1.6441850	2.1545220
H	-2.0156830	-1.8158520	1.2045230

H	-0.5240190	-2.6247840	0.6748520
C	-0.9302020	1.8615420	0.9271870
H	-2.0155810	1.9505090	0.9718240
H	-0.5231590	1.8965770	1.9360440
H	-0.5236550	2.6879250	0.3470890
Cl	2.0027710	-0.0001520	-0.0015610
Sn	-0.3789800	0.0000310	-0.0000140

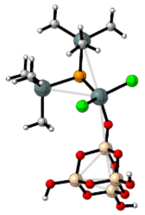

47

3Sn 0 1 scf done: -4058.183620 Sum of electronic and thermal Free Energies -4057.960675

Si	-1.9378570	0.3571180	0.6729590
Si	-4.5944830	-0.1249690	1.4745750
Si	-4.0852170	1.1478310	-0.9642350
Si	-3.4889710	-1.5785350	-0.6442810
O	-2.0163670	-1.1117830	-0.0924370
O	-3.0472920	0.2679410	1.8925920
O	-2.5627360	1.4422130	-0.4074790
O	-5.0483170	1.0105850	0.3630570
O	-4.5037520	-1.5518580	0.6599070
O	-4.0214930	-0.3650190	-1.6288420
O	-4.6530350	2.2811520	-1.9582760
H	-4.0891370	2.6071960	-2.6636470
O	-3.3815420	-3.0172390	-1.3630710
H	-4.1798470	-3.4384460	-1.6900010
O	-5.6031530	-0.2612040	2.7228570
H	-5.7614750	0.5009640	3.2846590
O	-0.4726810	0.7149850	1.1850740
Sn	1.2781560	0.8867780	0.2907820
Cl	1.7068310	3.1959270	0.3490030
Cl	0.7349920	0.4309250	-1.9595070
P	3.0175860	-0.4501340	1.3783390
C	3.3642140	-2.3556770	-2.0666590
H	3.2701640	-3.3180670	-2.5698280
H	2.7267560	-1.6271130	-2.5612950
H	4.4017130	-2.0312830	-2.1171410
C	4.0617010	-3.9514630	1.0184460
H	4.0010830	-4.9374360	0.5580240
H	5.0876030	-3.5926050	0.9562420
H	3.7716100	-4.0280560	2.0650190
C	0.6761800	-3.1924730	0.1512760
H	0.3873280	-3.2239110	1.2003610
H	0.0266910	-2.4959390	-0.3730140

H	0.5599580	-4.1855210	-0.2821670
C	4.5018620	1.3265850	-1.8209590
H	3.8317040	2.1821830	-1.7664020
H	5.4217580	1.6237800	-2.3245900
H	4.0228800	0.5303410	-2.3845910
C	6.5165360	-0.8727070	0.1471930
H	6.2396260	-1.6732680	-0.5361850
H	7.4598720	-0.4377200	-0.1820140
H	6.6459040	-1.2840790	1.1470260
C	5.5571080	2.3186000	1.4299520
H	4.7309300	3.0257420	1.4747200
H	5.7843650	1.9632020	2.4334870
H	6.4345750	2.8138730	1.0148730
Sn	4.9894150	0.6603740	0.1740460
Sn	2.7354990	-2.5889510	-0.0128970

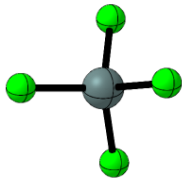

5
SnCl4 0 1 scf done: -2055.481706 Sum of electronic and thermal Free Energies -2055.512556

Sn	2.5996180	0.0019350	0.1490700
Cl	4.4170640	0.0039820	1.5735120
Cl	2.6744870	1.8751560	-1.1990530
Cl	2.6558250	-1.8920400	-1.1706260
Cl	0.6530440	0.0211560	1.3908780

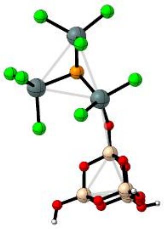

29
4Sn 0 1 scf done: -6580.215715 Sum of electronic and thermal Free Energies -6580.201394

Si	-1.9021400	0.1247470	0.6617970
Si	-4.5772560	-0.0599520	1.4877460
Si	-3.9550430	1.0959880	-0.9878060
Si	-3.6341150	-1.6726040	-0.6019590
O	-2.1168680	-1.3401580	-0.0652620
O	-2.9916760	0.1806150	1.8935830
O	-2.4025840	1.2482070	-0.4415190
O	-4.9085910	1.0936850	0.3520480
O	-4.6319450	-1.5047870	0.7046360
O	-4.0403270	-0.4306280	-1.6132790

O	-4.4156450	2.2571940	-2.0017950
H	-3.9339000	2.3928570	-2.8210230
O	-3.6753280	-3.1289240	-1.2839050
H	-4.5142710	-3.5031690	-1.5624410
O	-5.5870190	-0.0626990	2.7397080
H	-5.7049900	0.7356650	3.2595640
O	-0.3950240	0.3735600	1.1506120
Sn	1.2418370	0.8403450	0.1938020
Cl	1.6608130	3.0835850	0.6043650
Cl	0.8505160	0.4888620	-2.0619410
P	3.0411860	-0.5188200	1.3088570
Sn	2.9553450	-2.4699810	-0.2970990
Sn	5.0008010	0.6775890	0.2647590
Cl	5.7465510	2.2926930	1.7525240
Cl	6.6921850	-0.8856770	-0.0358330
Cl	4.5538560	1.7266590	-1.7553820
Cl	4.1552690	-4.2224820	0.6335530
Cl	3.8100960	-2.0567260	-2.4128020
Cl	0.7215630	-3.0609680	-0.4649460

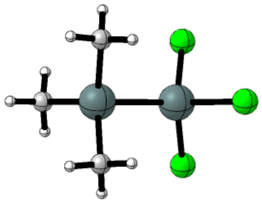

17

$SnMe_3SnCl_3$ 0 1 scf done: -1929.438814 Sum of electronic and thermal Free Energies -1929.378090

Cl	4.6915150	0.0307830	1.3569680
Cl	2.9138460	1.8426240	-1.3841410
Cl	2.9230540	-1.8920840	-1.3137500
C	-1.2690620	0.0253830	0.2230480
H	-2.2155060	0.0360000	0.7627610
H	-1.2025130	0.9090630	-0.4083100
H	-1.2157590	-0.8676110	-0.3963740
C	0.4783770	1.8303620	2.8110410
H	-0.3994390	1.9023690	3.4524450
H	1.3754690	1.8072780	3.4264060
H	0.5148110	2.6933000	2.1492520
C	0.4581430	-1.7752350	2.8273650
H	1.3512180	-1.7530820	3.4485960
H	-0.4243820	-1.8356900	3.4634700
H	0.4930660	-2.6440030	2.1731560
Sn	2.7085820	0.0016040	0.0775540
Sn	0.3553420	0.0228650	1.6413360

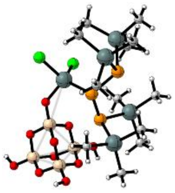

75

5Sn 0 1 scf done: -5409.415324 Sum of electronic and thermal Free Energies -5409.001828

Si	-2.9798320	-1.7517290	-0.8390590
Si	-4.5354020	0.5391430	-1.3881780
Si	-5.7935310	-1.8900370	-0.8351480
Si	-4.4527200	-0.5042820	1.2023170
O	-3.1151160	-1.3316530	0.7600760
O	-3.1967090	-0.3557570	-1.7086450
O	-4.3518850	-2.6273330	-1.1455950
O	-5.8067030	-0.4735430	-1.6774290
O	-4.5340310	0.8339800	0.2317850
O	-5.7364960	-1.4406050	0.7572200
O	-7.0770900	-2.7851770	-1.2175670
H	-7.0544140	-3.7292320	-1.0444270
O	-4.3886270	-0.1416520	2.7740430
H	-5.1251360	0.3227660	3.1786620
O	-4.5935640	1.9415930	-2.1841970
H	-4.5982090	1.9374060	-3.1442730
O	-1.6216970	-2.5019620	-1.1801510
Sn	0.2795980	-2.2043550	-0.6862260
Cl	1.3490920	-3.5909760	-2.2633140
Cl	0.4202490	-3.3559950	1.3762550
P	0.8894520	0.2333550	-0.8506880
P	2.7638500	0.6986430	0.1879360
P	-0.8508910	0.9578960	0.2462850
C	-2.6370350	4.2687910	-0.1958010
H	-2.8041140	5.1327210	-0.8388270
H	-3.4934890	3.6019950	-0.2630340
H	-2.5137440	4.6040400	0.8314680
C	0.9314810	4.3372580	-0.4070610
H	1.0485730	5.1684270	-1.1022760
H	0.8740130	4.7275490	0.6082580
H	1.7926350	3.6753920	-0.4806440
C	-0.9939440	2.7588190	-2.9729700
H	-0.0020020	2.5668730	-3.3763180
H	-1.6076920	1.8677670	-3.0926440
H	-1.4486730	3.5914440	-3.5091140
C	1.4333170	2.2448830	3.2460120
H	1.6490240	3.0679460	2.5683130
H	1.3608090	2.6204990	4.2672290
H	2.2364710	1.5175850	3.1801360
C	-2.0375980	2.7562940	3.1863030
H	-1.7238270	3.7744460	2.9620930

H	-2.9287550	2.5171650	2.6090940
H	-2.2709520	2.6823920	4.2484420
C	-0.8719760	-0.5164160	3.7125050
H	-0.3905560	-0.5297280	4.6896530
H	-1.9513970	-0.5947360	3.8285530
H	-0.5223800	-1.3591930	3.1222060
C	2.8263660	-1.3551630	3.4617960
H	1.7519820	-1.4498980	3.3412440
H	3.2041440	-2.2483580	3.9591600
H	3.0548680	-0.4820060	4.0697290
C	5.8032090	-0.4238020	1.8255150
H	5.7782710	0.6469630	2.0210460
H	6.2629360	-0.9294130	2.6747870
H	6.4048180	-0.6111550	0.9374820
C	3.9336180	-3.0961490	0.5235650
H	3.3769320	-3.8460230	1.0797340
H	3.5411270	-3.0446610	-0.4876590
H	4.9867690	-3.3732080	0.4792020
C	6.0334240	1.7449980	-1.3896480
H	5.7851420	2.7176340	-0.9674880
H	6.6145220	1.1808500	-0.6630990
H	6.6313860	1.8889150	-2.2894680
C	4.6692390	-1.2902290	-2.6422690
H	5.3017010	-1.8228940	-1.9345530
H	3.7517820	-1.8549360	-2.7962810
H	5.1969480	-1.1987870	-3.5916280
C	3.1003780	1.8188100	-3.3653080
H	2.7691710	2.7599910	-2.9287300
H	3.7362620	2.0275720	-4.2253780
H	2.2311730	1.2499130	-3.6883370
Sn	-0.4611040	1.3477920	2.7068390
Sn	-0.8698640	3.2234040	-0.8652880
Sn	4.2135430	0.6869850	-1.8962730
Sn	3.7952720	-1.1930050	1.5402180

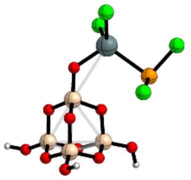

23

6Sn 0 1 scf done: -4310.247313 Sum of electronic and thermal Free Energies -4310.218442

Si	-1.0456080	-0.8329940	-0.4448350
Si	-3.4939730	0.0079080	-1.5500140
Si	-3.3174270	-0.5573830	1.1869430
Si	-2.0625900	1.7313590	0.1353440
O	-0.7426770	0.7837420	-0.1478940
O	-2.0915140	-0.8511900	-1.7145550

O	-1.9125900	-1.3584650	0.8540340
O	-4.2119040	-0.5971890	-0.1914010
O	-3.0419630	1.5470430	-1.1772480
O	-2.8661540	1.0216990	1.3873420
O	-4.1518790	-1.1688030	2.4181790
H	-3.7158500	-1.3353340	3.2569680
O	-1.5809750	3.2404330	0.4166230
H	-2.2278100	3.9391430	0.5392900
O	-4.4312090	0.0061800	-2.8556510
H	-4.7528700	-0.8236780	-3.2155230
O	0.3401960	-1.5903720	-0.6757250
Sn	2.1174020	-0.7515200	-0.4111760
Cl	2.8787170	-0.2457950	-2.5562070
Cl	3.4234800	-2.4649050	0.4629250
P	1.8932630	1.3232410	1.1115400
Cl	3.8832190	1.3689090	1.7088150
Cl	1.0052890	0.3076900	2.6878230

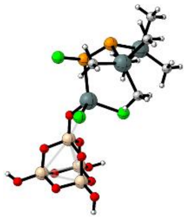

49

7Sn 0 1 scf done: -4859.825476 Sum of electronic and thermal Free Energies -4859.606118

Si	2.4579080	-0.0167480	-0.6718500
Si	5.1664140	-0.7486100	-0.9033950
Si	4.4878180	1.8648230	-0.1807070
Si	4.0583060	-0.2498420	1.6208250
O	2.5845430	-0.4364170	0.9250440
O	3.6179650	-0.8941870	-1.4541730
O	2.9739500	1.5521900	-0.7554440
O	5.5088930	0.8660450	-0.9959440
O	5.1242090	-1.1154040	0.6995540
O	4.4771070	1.3317170	1.3833410
O	4.9553350	3.3979190	-0.3500110
H	4.4373160	4.0929390	0.0623530
O	4.0192870	-0.6980250	3.1670710
H	4.8440390	-0.7979300	3.6479050
O	6.2313100	-1.6894460	-1.6614700
H	6.3415380	-1.6052160	-2.6113240
O	0.9875610	-0.2404180	-1.2501640
Sn	-0.7587210	-0.5077820	-0.3630340
Cl	-0.7792290	1.1747130	1.2973130
Cl	-0.5681280	-2.5734450	0.7466660
P	-2.6688390	-0.2691300	-2.0611990

P	-4.5314830	-0.0977050	-0.9857740
C	-4.3800590	-3.5549110	0.4931000
H	-3.3386520	-3.7028950	0.2184500
H	-4.6395970	-4.2267860	1.3109280
H	-5.0162990	-3.7616100	-0.3649190
C	-6.7187570	-1.1317050	1.7039140
H	-7.3873530	-1.3859500	0.8831460
H	-6.9768090	-1.7335950	2.5750740
H	-6.8460540	-0.0792130	1.9544350
C	-3.3651130	-0.9774140	2.7700870
H	-2.3557400	-1.3400090	2.6012590
H	-3.3485280	0.0987090	2.9117040
H	-3.7722960	-1.4528350	3.6633180
C	-6.1940710	3.1974140	-1.1330880
H	-6.2255700	3.0528720	-2.2117430
H	-7.0513020	2.6985300	-0.6833470
H	-6.2445180	4.2634790	-0.9116820
C	-4.3386310	2.5888740	1.8079560
H	-5.1141160	1.9724910	2.2593290
H	-3.3660700	2.2987230	2.1961600
H	-4.5303870	3.6332720	2.0534920
C	-2.6539300	3.2849900	-1.2900710
H	-2.6272360	3.0280980	-2.3472190
H	-2.7452240	4.3664540	-1.1895640
H	-1.7354510	2.9597530	-0.8075280
Cl	-2.6536900	-2.2935240	-2.6082220
Sn	-4.3625980	2.3683590	-0.3388190
Sn	-4.6655370	-1.5170550	1.1341580

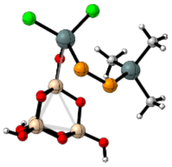

35

8Sn 0 1 scf done: -4065.331832 Sum of electronic and thermal Free Energies -4065.203894

Si	-0.8931630	-0.3964380	0.4781240
Si	-2.6762450	0.9092560	-1.2935950
Si	-3.1239930	0.9964880	1.4724840
Si	-0.9836560	2.4169720	0.3389140
O	-0.0827460	1.0579390	0.5416780
O	-1.6759090	-0.3651580	-0.9853870
O	-2.0754080	-0.2698910	1.6192170
O	-3.7558160	0.9149040	-0.0436970
O	-1.7528940	2.2605310	-1.1112150
O	-2.1695900	2.3483690	1.4812200
O	-4.2971380	1.0082790	2.5731070
H	-4.0881920	0.8300100	3.4929420

O	-0.0274780	3.7114710	0.4369160
H	-0.3632180	4.5719170	0.1743270
O	-3.3499340	0.8919570	-2.7538630
H	-3.9693230	0.1961180	-2.9860660
O	0.0609550	-1.6577650	0.6267500
Sn	1.0966110	-2.5560540	-0.8368650
Cl	-0.0988360	-4.5538010	-1.1614990
Cl	3.0642500	-3.1556650	0.3223320
P	1.3158620	-0.7937740	-2.6963100
P	2.1718440	0.8094910	-1.8655280
C	2.4444230	-0.0281580	2.2394320
H	2.0374330	-1.0160510	2.0591290
H	3.2419960	-0.0898760	2.9803840
H	1.6613450	0.6400510	2.5870640
C	3.4686080	2.9690540	0.6805410
H	2.4706740	3.3950130	0.7617860
H	4.0380090	3.2020410	1.5804830
H	3.9764010	3.3940450	-0.1839110
C	5.2466890	-0.0233470	-0.0331290
H	5.9183880	0.1070020	0.8154650
H	5.1341620	-1.0833240	-0.2464180
H	5.6610500	0.4872340	-0.9006260
Sn	3.3196960	0.8248050	0.4688050

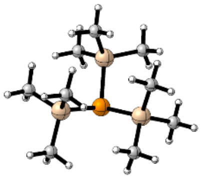

40

$P(SiMe_3)_3$ 0 1 scf done: -1569.555125 Sum of electronic and thermal Free Energies -1569.268731

P	2.7841910	0.1839580	-0.0046890
Si	3.8594910	0.2827210	1.9763920
Si	2.5326170	2.3430890	-0.6082630
C	0.9910190	2.9810870	0.2579430
H	0.7907800	4.0153890	-0.0347740
H	0.1217320	2.3776940	-0.0095450
H	1.0951140	2.9533110	1.3428910
C	3.9761040	3.4749680	-0.1846670
H	3.7674890	4.4869000	-0.5434340
H	4.1404100	3.5344600	0.8917940
H	4.9032120	3.1383660	-0.6486640
C	2.2371190	2.4149060	-2.4650630
H	3.1277750	2.1505910	-3.0358560
H	1.4323380	1.7404690	-2.7622800
H	1.9483120	3.4297160	-2.7514980
C	2.9392710	1.4819800	3.0968820
H	1.8759100	1.2411010	3.1429540

H	3.3424450	1.4191680	4.1111830
H	3.0374890	2.5160690	2.7651700
C	5.6661590	0.8039220	1.8922410
H	5.7824470	1.7872760	1.4367480
H	6.0829110	0.8487050	2.9024870
H	6.2649620	0.0943900	1.3201290
C	3.7608540	-1.4313430	2.7419680
H	2.7209260	-1.7425850	2.8558900
H	4.2671840	-2.1780880	2.1298090
H	4.2273090	-1.4337800	3.7308900
C	5.7160950	0.5778730	-1.8858210
H	6.4227690	0.0854070	-2.5598750
H	5.3361560	1.4631500	-2.3969070
H	6.2663060	0.9078480	-1.0047100
C	3.4245620	-1.1048760	-3.0107270
H	2.9474040	-0.2474730	-3.4860860
H	4.1203530	-1.5449420	-3.7302810
H	2.6484950	-1.8412140	-2.7942870
C	5.0930390	-2.1670950	-0.6922300
H	5.7179220	-1.9457250	0.1735400
H	4.3218080	-2.8737090	-0.3810510
H	5.7215880	-2.6620450	-1.4373720
Si	4.3316920	-0.6166220	-1.4382230

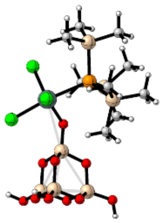

61

2Si 0 1 scf done: -5078.209609 Sum of electronic and thermal Free Energies -5077.865018

Si	1.9460130	0.0349760	0.4566490
Si	3.8790890	0.1388150	-1.5889410
Si	4.6350830	0.5755300	1.0809700
Si	3.8981490	-1.9842730	0.2337050
O	2.3534150	-1.5700280	0.6067780
O	2.3304640	0.4201550	-1.1066880
O	3.0398190	0.8272550	1.4038390
O	4.8289660	0.9566940	-0.5147450
O	4.1596150	-1.4605600	-1.3111710
O	4.8761720	-1.0513200	1.1815800
O	5.6380950	1.3521960	2.0736500
H	5.4937480	2.2863070	2.2429870
O	4.1055190	-3.5707730	0.4312800
H	4.9776220	-3.9486140	0.2955540
O	4.1588710	0.4964380	-3.1347520
H	3.8957130	1.3592520	-3.4646410

O	0.4083640	0.2652460	0.7996370
Sn	-0.9035670	1.5875460	0.0289780
Cl	0.7251280	3.3215340	0.1276080
Cl	-2.3999720	2.7270590	1.4899830
P	-2.4753820	-0.5396250	0.0278220
Si	-2.1134820	-1.7192420	1.9637900
Si	-1.9729090	-1.8986900	-1.7522740
C	-0.1189310	-1.9287600	-1.9318490
H	0.1493900	-2.6106740	-2.7434990
H	0.2764760	-0.9444260	-2.1812740
H	0.3787320	-2.2740240	-1.0266330
C	-2.6490260	-3.6086980	-1.3743310
H	-2.4414980	-4.2576790	-2.2297380
H	-2.1846260	-4.0601560	-0.4990850
H	-3.7282010	-3.5957830	-1.2230590
C	-2.8015370	-1.2485700	-3.2976310
H	-3.8883190	-1.2420700	-3.2183670
H	-2.4634830	-0.2421770	-3.5353850
H	-2.5347050	-1.9049200	-4.1307850
C	-0.6114470	-2.8131670	1.8025280
H	-0.4712910	-3.3286480	2.7574490
H	-0.7302880	-3.5780630	1.0358740
H	0.2943460	-2.2531380	1.5879140
C	-3.6175350	-2.8071080	2.2532950
H	-3.7724760	-3.5048610	1.4299140
H	-3.4475120	-3.3973050	3.1581040
H	-4.5349000	-2.2389690	2.3984760
C	-1.9051130	-0.4691190	3.3330200
H	-0.9919680	0.1080820	3.1875370
H	-2.7410520	0.2271020	3.3903350
H	-1.8276570	-0.9929730	4.2895610
Cl	-1.2339260	1.8545870	-2.3171680
C	-5.6634290	-1.4376810	-0.6636110
H	-6.7223950	-1.1669450	-0.6963340
H	-5.3784830	-1.7747110	-1.6592440
H	-5.5569250	-2.2748430	0.0261510
C	-4.8057870	1.4528040	-1.3331650
H	-4.3850890	1.1812980	-2.3005900
H	-5.8574590	1.7152480	-1.4768680
H	-4.2812170	2.3388320	-0.9757410
C	-5.3029250	0.5926700	1.5843100
H	-5.2642030	-0.2106310	2.3190950
H	-4.7365970	1.4399000	1.9649090
H	-6.3482380	0.8981130	1.4829150
Si	-4.6833200	0.0595880	-0.0974430

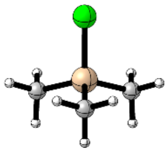

14

ClSiMe₃ 0 1 scf done: -869.659245 Sum of electronic and thermal Free Energies -869.579574

Si	2.6571190	-2.1937420	-1.3627370
C	4.2444820	-2.1305040	-2.3422240
H	4.3337310	-3.0145150	-2.9791860
H	4.2749970	-1.2468030	-2.9817290
H	5.1118220	-2.0997230	-1.6807130
C	2.6248390	-3.6603510	-0.2090320
H	1.7139490	-3.6659190	0.3918580
H	2.6627040	-4.5930390	-0.7780560
H	3.4784870	-3.6423470	0.4705370
C	1.1633100	-2.1709500	-2.4812270
H	1.1543460	-3.0564010	-3.1223360
H	0.2394680	-2.1628800	-1.9004690
H	1.1678720	-1.2877410	-3.1221420
Cl	2.5808470	-0.4681870	-0.1826350

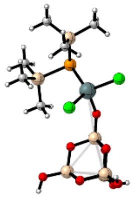

47

3Si 0 1 scf done: -4208.539059 Sum of electronic and thermal Free Energies -4208.300897

Si	-0.6826900	-1.3586280	-1.5989770
Si	-3.0656280	-1.1013300	-0.1213190
Si	-1.0986930	-2.9085030	0.7115900
Si	-0.6571680	-0.1426740	0.9315450
O	-0.0597880	-0.1952670	-0.5956040
O	-2.3144910	-1.1088420	-1.5907920
O	-0.4536730	-2.7883480	-0.8000330
O	-2.7001760	-2.5605790	0.5629530
O	-2.2939310	0.0352530	0.7839780
O	-0.4447730	-1.6524760	1.5651870
O	-0.8967660	-4.3503550	1.4007630
H	-0.0302680	-4.7629980	1.3734960
O	0.0570480	1.0349240	1.7684600
H	-0.2309720	1.2127350	2.6668060
O	-4.6433170	-0.7839450	-0.1859840
H	-5.2229990	-1.3778590	-0.6685040
O	-0.0537760	-1.3228670	-3.0623300
Sn	1.7698160	-1.5614540	-3.7727740

Cl	1.7352600	-3.7039630	-4.7236300
Cl	3.0529060	-1.7949390	-1.8124330
P	2.3099850	0.2174400	-5.3721770
Si	4.2311820	-0.6806570	-6.2375100
Si	2.9775770	1.8371220	-3.8923380
C	4.6090580	1.4969590	-3.0384830
H	4.8378820	2.3302250	-2.3681080
H	4.5606640	0.5886360	-2.4382660
H	5.4344580	1.4037950	-3.7446310
C	3.1151100	3.3666900	-4.9712680
H	3.3906980	4.2272880	-4.3559000
H	3.8742230	3.2528640	-5.7460890
H	2.1645080	3.5904540	-5.4580560
C	1.6048720	2.0353520	-2.6391640
H	0.6308880	2.1236430	-3.1231190
H	1.5514590	1.2078480	-1.9311060
H	1.7742090	2.9461380	-2.0582240
C	5.2603610	-1.6431150	-5.0030490
H	4.7486390	-2.5497400	-4.6756810
H	6.1947180	-1.9511280	-5.4810500
H	5.5094470	-1.0580480	-4.1190930
C	5.2001840	0.7733650	-6.9234920
H	5.5840800	1.4209090	-6.1348960
H	6.0549000	0.4014210	-7.4945590
H	4.5889650	1.3787780	-7.5949590
C	3.6758480	-1.8079570	-7.6238470
H	3.0613980	-2.6239560	-7.2405230
H	3.0917770	-1.2626040	-8.3669740
H	4.5433130	-2.2453950	-8.1251780

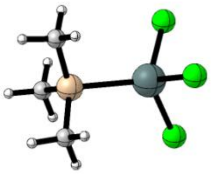

17

$SnCl_3SiMe_3$ 0 1 scf done: -2004.610508 Sum of electronic and thermal Free Energies -2004.541945

Sn	2.5365260	0.0028840	0.1944610
Cl	4.5135390	0.0026820	1.4756860
Cl	2.7387760	1.8613250	-1.2388690
Cl	2.7219900	-1.8765270	-1.2135740
Si	0.3702790	0.0224180	1.6333590
C	-1.0414070	0.0197130	0.4100580
H	-1.9951140	0.0275960	0.9452230
H	-1.0084180	0.8990980	-0.2347670
H	-1.0159430	-0.8690370	-0.2221440
C	0.4633270	1.5887660	2.6469280
H	-0.4205060	1.6695040	3.2859360

H	1.3457900	1.5933050	3.2883790
H	0.5004600	2.4723640	2.0081130
C	0.4499900	-1.5300630	2.6691630
H	1.3324350	-1.5330270	3.3106480
H	-0.4344530	-1.5941150	3.3092170
H	0.4794920	-2.4229620	2.0430070

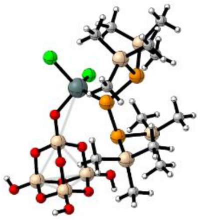

75

5Si 0 1 scf done: -5710.123838Sum of electronic and thermal Free Energies -5709.679438

Si	2.4756860	-1.2664020	0.5761420
Si	4.0189800	0.9934090	1.2666130
Si	5.2921040	-1.3757450	0.5236770
Si	3.9061720	0.1372390	-1.3902530
O	2.5787490	-0.7282460	-0.9896830
O	2.6877820	0.0694560	1.5348810
O	3.8620580	-2.1452590	0.8000020
O	5.3035340	-0.0246230	1.4666260
O	3.9866400	1.4030260	-0.3276290
O	5.2061740	-0.8138510	-1.0311800
O	6.5900390	-2.2821790	0.8219770
H	6.5751620	-3.2108840	0.5790570
O	3.8196940	0.6027200	-2.9327580
H	4.5834290	1.0141130	-3.3439610
O	4.0861870	2.3302480	2.1672420
H	4.2411160	2.2525310	3.1115240
O	1.1412020	-2.0699070	0.8833250
Sn	-0.7392120	-1.9349800	0.2705330
Cl	-1.7844270	-3.4770910	1.7061170
Cl	-0.6213080	-3.0125690	-1.8253060
P	-1.5295800	0.3959230	0.6818300
P	-3.5333750	0.8114390	-0.1078180
P	0.1176410	1.4359930	-0.2991540
Si	0.0640200	3.3098100	0.9733300
Si	-0.2234310	1.9487590	-2.4770750
Si	-4.6803060	0.6510560	1.8563320
Si	-4.4213920	-0.7792730	-1.4727100
C	1.5390410	4.3621090	0.4932770
H	1.6288100	5.1917010	1.2001300
H	2.4666530	3.7914690	0.5300100
H	1.4358620	4.7848440	-0.5052770
C	-1.5256200	4.2879030	0.7637700
H	-1.5780610	5.0956720	1.4988790

H	-1.5811880	4.7343970	-0.2303000
H	-2.4041680	3.6535950	0.8870020
C	0.2571010	2.7261400	2.7442740
H	-0.6188960	2.1792100	3.0913570
H	1.1231460	2.0692960	2.8328690
H	0.4045630	3.5864370	3.4025620
C	-1.8799110	2.7427310	-2.8504920
H	-1.9531620	3.7219460	-2.3753030
H	-1.9882450	2.8797730	-3.9301720
H	-2.7133710	2.1434070	-2.4909820
C	1.1480510	3.1525780	-2.9241650
H	0.9499790	4.1567500	-2.5498150
H	2.1141240	2.8226290	-2.5418610
H	1.2230030	3.2111640	-4.0135990
C	0.0406900	0.3599020	-3.4272270
H	-0.2073840	0.5054740	-4.4818610
H	1.0869800	0.0602480	-3.3579270
H	-0.5574300	-0.4677470	-3.0527480
C	-3.6596640	-0.6128200	-3.1739990
H	-2.5976330	-0.8512430	-3.1662440
H	-4.1480350	-1.3210520	-3.8490790
H	-3.7852800	0.3890630	-3.5844110
C	-6.2425680	-0.3167570	-1.5516580
H	-6.3901070	0.7463930	-1.7456750
H	-6.7132000	-0.8763100	-2.3645690
H	-6.7655000	-0.5732790	-0.6298850
C	-4.2732860	-2.5581130	-0.9052600
H	-3.3024220	-2.9899960	-1.1418260
H	-4.4581600	-2.6909370	0.1579120
H	-5.0198110	-3.1422340	-1.4524960
C	-6.2743750	1.6122870	1.6008990
H	-6.0611340	2.6428370	1.3113410
H	-6.9103280	1.1726460	0.8332840
H	-6.8429710	1.6346790	2.5345210
C	-5.0538460	-1.1013980	2.4062870
H	-5.7499910	-1.5998450	1.7306720
H	-4.1468140	-1.7049630	2.4653180
H	-5.5112590	-1.0834070	3.3993960
C	-3.6646480	1.5151160	3.1767510
H	-3.3518920	2.5113770	2.8610510
H	-4.2736820	1.6239020	4.0783320
H	-2.7727900	0.9461400	3.4375110

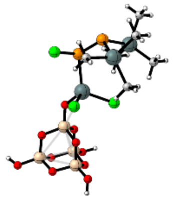

49

7Si 0 1 scf done: -5010.181935 Sum of electronic and thermal Free Energies -5009.948357

Si	-2.5000050	-1.4492870	-0.3535350
Si	-4.8474550	-0.4774060	-1.5628920
Si	-4.4027590	-0.0343970	1.1635910
Si	-2.8518760	1.3137460	-0.7519590
O	-1.8305980	0.0289510	-0.7146320
O	-3.7049400	-1.6654660	-1.4579690
O	-3.2662360	-1.2268300	1.0945490
O	-5.4780860	-0.3455990	-0.0414220
O	-4.0325790	0.9249500	-1.8402760
O	-3.6093740	1.3454210	0.7141870
O	-5.1604470	0.0730730	2.5804950
H	-4.6456090	0.0651170	3.3907040
O	-2.0376820	2.6567130	-1.1100520
H	-2.5080780	3.4909740	-1.1753490
O	-5.9466340	-0.7188070	-2.7139320
H	-6.4772590	-1.5187300	-2.7002900
O	-1.3903090	-2.5923270	-0.3413370
Sn	0.5471920	-2.4145450	0.0617320
Cl	1.5125240	-3.8996380	-1.4904310
Cl	0.6877150	-3.4244010	2.1833740
P	1.3501200	0.0102270	-0.2364740
P	3.5001090	0.0932940	-0.0342190
Si	4.1388790	-0.4484470	-2.1636070
Si	4.4410160	-1.3183380	1.4875740
C	3.6574220	-0.9221010	3.1377650
H	2.6159020	-1.2418560	3.1681630
H	4.1939520	-1.4508040	3.9303010
H	3.6929940	0.1465410	3.3516470
C	6.2441330	-0.7935290	1.4406190
H	6.3552200	0.2672220	1.6702280
H	6.8169060	-1.3625520	2.1775390
H	6.6916080	-0.9768370	0.4618130
C	4.3188170	-3.1498740	1.1270080
H	3.2976240	-3.5241680	1.1322720
H	4.7797270	-3.4182760	0.1790790
H	4.8533290	-3.6768980	1.9236560
C	5.4089840	0.8571790	-2.6077280
H	4.9725450	1.8567310	-2.5772730
H	6.2513820	0.8395500	-1.9132010
H	5.7989200	0.6832690	-3.6141190

C	4.9192820	-2.1455000	-2.2821530
H	5.7948560	-2.2406980	-1.6388910
H	4.2096280	-2.9317070	-2.0277480
H	5.2477810	-2.3074210	-3.3127640
C	2.6565520	-0.3478240	-3.3057290
H	2.1310680	0.6042600	-3.2268690
H	3.0034330	-0.4553600	-4.3373370
H	1.9443900	-1.1524390	-3.1156720
Cl	0.6984850	0.6289560	1.6516320

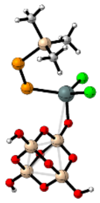

35

8Si 0 1 scf done: -4140.500747 Sum of electronic and thermal Free Energies -4140.369673

Si	-2.5000050	-1.4492870	-0.3535350
Si	-2.2747710	-0.7610310	-0.9258190
Si	-4.1535390	1.3065970	-0.5407220
Si	-4.5328480	-1.1733790	0.6965500
Si	-2.4770410	0.5395750	1.5672010
O	-1.5146430	-0.1738040	0.4416470
O	-3.0984710	0.5293320	-1.5434700
O	-3.4357410	-1.7913650	-0.3698800
O	-5.2055220	0.1391620	-0.0308540
O	-3.2832580	1.7491150	0.7849320
O	-3.6303660	-0.5765980	1.9480390
O	-5.6733550	-2.2171090	1.1445370
H	-5.4225360	-3.1067270	1.4041210
O	-1.5866190	1.0389080	2.8133770
H	-1.9968050	1.5571260	3.5097320
O	-4.8628450	2.5959440	-1.1922330
H	-5.4029960	2.4930780	-1.9791820
O	-1.1825760	-1.3954010	-1.8965370
Sn	0.7512400	-1.4560320	-1.3908130
Cl	1.7400380	-2.3043770	-3.3440270
Cl	0.7648020	-3.2372900	0.1583680
P	1.2748960	0.9924140	-0.7803190
P	2.8873140	1.2686040	0.3728570
Si	4.0270180	-0.6388150	0.9884310
C	3.1039590	-1.3596150	2.4457370
H	2.1048120	-1.6889960	2.1634160
H	3.6488320	-2.2267370	2.8292100
H	3.0154740	-0.6308580	3.2530320
C	5.6925330	0.0439430	1.5142340
H	5.5864270	0.8015230	2.2926210

H	6.3167750	-0.7613960	1.9101460
H	6.2229630	0.4952190	0.6735190
C	4.2519270	-1.8445050	-0.4255710
H	3.4178190	-2.5348160	-0.5359070
H	4.4193560	-1.3418950	-1.3784740
H	5.1366660	-2.4511230	-0.2114720

References

[1] R. Zazpe, J. Charvot, J. Rodriguez-Pereira, L. Hromádko, M. Kurka, K. Baishya, H. Sopha, F. Bureš, J. M. Macak, "Synthesis of titanium phosphide by thermal ALD based on a novel phosphorus precursor" *Nanoscale* **2025**, *17*, 12406–12415.

[2] J. Frisch, G. W. Trucks, H. B. Schlegel, G. E. Scuseria, M. A. Robb, J. R. Cheeseman, G. Scalmani, V. Barone, G. A. Petersson, H. Nakatsuji, X. Li, M. Caricato, A. V. Marenich, J. Bloino, B. G. Janesko, R. Gomperts, B. Mennucci, H. P. Hratchian, J. V. Ortiz, A. F. Izmaylov, J. L. Sonnenberg, D. Williams-Young, F. Ding, F. Lipparini, F. Egidi, J. Goings, B. Peng, A. Petrone, T. Henderson, D. Ranasinghe, V. G. Zakrzewski, J. Gao, N. Rega, G. Zheng, W. Liang, M. Hada, M. Ehara, K. Toyota, R. Fukuda, J. Hasegawa, M. Ishida, T. Nakajima, Y. Honda, O. Kitao, H. Nakai, T. Vreven, K. Throssell, Jr. Montgomery, J. A., J. E. Peralta, F. Ogliaro, M. J. Bearpark, J. J. Heyd, E. N. Brothers, K. N. Kudin, V. N. Staroverov, T. A. Keith, R. Kobayashi, J. Normand, K. Raghavachari, A. P. Rendell, J. C. Burant, S. S. Iyengar, J. Tomasi, M. Cossi, J. M. Millam, M. Klene, C. Adamo, R. Cammi, J. W. Ochterski, R. L. Martin, K. Morokuma, O. Farkas, J. B. Foresman, D. J. Fox, "Gaussian 16" *Gaussian 16, Revision A.03 M* **2016**, Wallingford, Gaussian Inc.

[3] A. D. Becke, "Density-functional thermochemistry. III. The role of exact exchange" *The Journal of Chemical Physics* **1993**, *98*, 5648–5652.

[4] C. Lee, W. Yang, R. G. Parr, "Development of the Colle-Salvetti correlation-energy formula into a functional of the electron density" *Physical Review B* **1988**, *37*, 785–789.

[5] S. Grimme, S. Ehrlich, L. Goerigk, "Effect of the damping function in dispersion corrected density functional theory" *Journal of Computational Chemistry* **2011**, *32*, 1456–1465.

[6] F. Weigend, R. Ahlrichs, "Balanced basis sets of split valence, triple zeta valence and quadruple zeta valence quality for H to Rn: Design and assessment of accuracy" *Physical Chemistry Chemical Physics* **2005**, *7*, 3297.
